# Comparative analyses define differences between BHD-associated renal tumour and sporadic chromophobe renal cell carcinoma

**DOI:** 10.1016/j.ebiom.2023.104596

**Published:** 2023-05-12

**Authors:** Ryosuke Jikuya, Todd A. Johnson, Kazuhiro Maejima, Jisong An, Young-Seok Ju, Hwajin Lee, Kyungsik Ha, WooJeung Song, Youngwook Kim, Yuki Okawa, Shota Sasagawa, Yuki Kanazashi, Masashi Fujita, Seiya Imoto, Taku Mitome, Shinji Ohtake, Go Noguchi, Sachi Kawaura, Yasuhiro Iribe, Kota Aomori, Tomoyuki Tatenuma, Mitsuru Komeya, Hiroki Ito, Yusuke Ito, Kentaro Muraoka, Mitsuko Furuya, Ikuma Kato, Satoshi Fujii, Haruka Hamanoue, Tomohiko Tamura, Masaya Baba, Toshio Suda, Tatsuhiko Kodama, Kazuhide Makiyama, Masahiro Yao, Brian M. Shuch, Christopher J. Ricketts, Laura S. Schmidt, W. Marston Linehan, Hidewaki Nakagawa, Hisashi Hasumi

**Affiliations:** a Department of Urology, Yokohama City University Graduate School of Medicine, 3-9 Fuku-ura, Kanazawa-ku, Yokohama, Kanagawa, 236-0004, Japan; b Laboratory for Cancer Genomics, RIKEN Center for Integrative Medical Sciences, Yokohama, Kanagawa, 230-0045, Japan; c Graduate School of Medical Science and Engineering (GSMSE), Korea Advanced Institute of Science and Technology (KAIST), Daejeon, Republic of Korea; d Biomedical Knowledge Engineering Laboratory, Seoul National University, Seoul, 08826, Republic of Korea; e UPPThera, Inc. BRC Laboratory 1-204 9, Songdomirae-ro, Yeonsu-gu, Incheon, Republic of Korea; f National Cancer Center Korea, 323 Ilsan-ro, Ilsandong-gu, Goyang-si Gyeonggi-do, Republic of Korea; g Human Genome Center, Institute of Medical Science, University of Tokyo, Minato-ku, Tokyo, Japan; h Pathology Center, GeneticLab Co., Ltd., 28-196, N9, W15, Chuo-ku, Sapporo, 060-0009, Japan; i Department of Molecular Pathology, Yokohama City University Graduate School of Medicine, 3-9 Fuku-ura, Kanazawa-ku, Yokohama, Kanagawa, 236-0004, Japan; j Clinical Genetics Department, Yokohama City University Graduate School of Medicine, Yokohama, Kanagawa, 236-0004, Japan; k Department of Immunology, Yokohama City University Graduate School of Medicine, 3-9 Fuku-ura, Kanazawa-ku, Yokohama, Kanagawa, 236-0004, Japan; l Advanced Medical Research Center, Yokohama City University Graduate School of Medicine, Yokohama, Kanagawa, 236-0004, Japan; m Laboratory of Cancer Metabolism, International Research Center for Medical Sciences, Kumamoto University, Kumamoto, 860-0811, Japan; n Laboratory for Systems Biology and Medicine, Research Center for Advanced Science and Technology, University of Tokyo, Tokyo, 153-8904, Japan; o Institute of Urologic Oncology, UCLA School of Medicine, Los Angeles, CA90095, USA; p Urologic Oncology Branch, Center for Cancer Research, National Cancer Institute, National Institutes of Health, Bethesda, MD20892, USA; q Basic Science Program, Frederick National Laboratory for Cancer Research, Frederick, MD, 21702, USA

**Keywords:** Birt-Hogg-Dubé (BHD) syndrome, Chromophobe renal cell carcinoma (ChRCC), Folliculin *(FLCN)*, Renal tumour predisposition syndrome

## Abstract

**Background:**

Birt-Hogg-Dubé (BHD) syndrome, caused by germline alteration of folliculin *(FLCN)* gene, develops hybrid oncocytic/chromophobe tumour (HOCT) and chromophobe renal cell carcinoma (ChRCC), whereas sporadic ChRCC does not harbor *FLCN* alteration. To date, molecular characteristics of these similar histological types of tumours have been incompletely elucidated.

**Methods:**

To elucidate renal tumourigenesis of BHD-associated renal tumours and sporadic renal tumours, we conducted whole genome sequencing (WGS) and RNA-sequencing (RNA-seq) of sixteen BHD-associated renal tumours from nine unrelated BHD patients, twenty-one sporadic ChRCCs and seven sporadic oncocytomas. We then compared somatic mutation profiles with *FLCN* variants and RNA expression profiles between BHD-associated renal tumours and sporadic renal tumours.

**Findings:**

RNA-seq analysis revealed that BHD-associated renal tumours and sporadic renal tumours have totally different expression profiles. Sporadic ChRCCs were clustered into two distinct clusters characterized by *L1CAM* and *FOXI1* expressions, molecular markers for renal tubule subclasses. Increased mitochondrial DNA (mtDNA) copy number with fewer variants was observed in BHD-associated renal tumours compared to sporadic ChRCCs. Cell-of-origin analysis using WGS data demonstrated that BHD-associated renal tumours and sporadic ChRCCs may arise from different cells of origin and second hit *FLCN* alterations may occur in early third decade of life in BHD patients.

**Interpretation:**

These data further our understanding of renal tumourigenesis of these two different types of renal tumours with similar histology.

**Funding:**

This study was supported by 10.13039/501100001691JSPS KAKENHI Grants, 10.13039/501100006264RIKEN internal grant, and the Intramural Research Program of the 10.13039/100000002National Institutes of Health (10.13039/100000002NIH), 10.13039/100000054National Cancer Institute (NCI), Center for Cancer Research.

**Research in context**

**Evidence before this study**

In our previous whole exome sequencing (WES) study, very few variants were commonly observed in BHD-associated renal tumour. Transcriptomic intra-tumour heterogeneity was observed in our single-cell RNA sequencing (scRNA-seq) study of BHD-associated renal tumour. To date, there is no study comparing BHD-associated renal tumourigenesis with renal tumourigenesis of sporadic ChRCC.

**Added value of this study**

Some molecular characteristics in common were observed between BHD-associated renal tumour and sporadic ChRCCs in bulk tissue or at single-cell level including heterogeneous molecular characteristics conferred by mutually exclusive expressions of *L1CAM* and *FOXI1*, distinguishing markers for principal cell and intercalated cell, respectively. BHD-associated renal tumours harbor mitochondrial DNA (mtDNA) with higher copy numbers and fewer variants compared to sporadic ChRCCs. BHD-associated renal tumours and sporadic ChRCCs may arise from different cells of origin and second hit *FLCN* alterations may occur in early third decade of life in BHD patients.

**Implications of all the available evidence**

Inter-tumour or intra-tumour heterogeneity in BHD-associated renal tumours and sporadic ChRCCs may be associated with the normal nephron developmental machinery. This study denotes that these two histologically similar renal tumours are different types of tumours arising from different cells of origin and distinct therapeutical approaches are needed for these tumours.

## Introduction

Birt-Hogg-Dubé (BHD) syndrome is a renal tumour predisposition syndrome, in which affected individuals develop a variety of histological types of renal tumours, pulmonary cysts and cutaneous fibrofolliculomas.^1^, ^2^, ^3^, ^4^ The folliculin (*FLCN*) gene, which is responsible for BHD syndrome, is a metabolic gene which regulates oxidative phosphorylation, amino acid sensing and kidney cell growth rate in collaboration with its interacting partners, folliculin-interacting proteins 1 (*FNIP1*) and 2 (*FNIP2*).^5^, ^6^, ^7^, ^8^, ^9^

The most frequent histology of BHD-associated renal tumour is either hybrid oncocytic/chromophobe tumour (HOCT) or chromophobe renal cell carcinoma (ChRCC). Although some of the pathological observations including intratumour peripheral small papillary tufts or strong GPNMB staining in cytoplasm have been reported to distinguish BHD-associated renal tumour from sporadic ChRCC, renal tumourigenesis of these histologically similar types of tumours remains elusive.^10^^,^^11^ BHD-associated renal tumourigenesis is triggered by biallelic loss of the *FLCN* gene, whereas sporadic ChRCC frequently harbors tumour protein p53 (*TP53*) and/or phosphatase and tensin homolog (*PTEN*) alterations without *FLCN* alteration and in classical types generally has copy loss of chromosomes 1, 2, 6, 10, 13 and 17.^1^^,^^12^, ^13^, ^14^, ^15^, ^16^ However, these tumours share some molecular features including increased oxidative phosphorylation with upregulation of PPARG coactivator 1 alpha (*PPARGC1a*), an important co-activator for mitochondrial metabolism, as well as increased forkhead box I1 (*FOXI1*) expression, a transcription factor critical for collecting duct differentiation, suggesting that these shared molecular features may lead to similar histologies in these genetically different tumours.^5^^,^^17^^,^^18^

Renal tumours do not develop as a result of a single gene alteration; however, our previous whole exome sequencing (WES) study of twenty-nine BHD-associated renal tumours demonstrated that very few genes were commonly mutated in BHD-associated renal tumour except for *FLCN* alteration.^16^ The lack of commonly mutated genes in BHD-associated renal tumours in addition to *FLCN* implies that an underlying molecular mechanism may trigger BHD-associated renal tumourigenesis.

To elucidate renal tumourigenesis and compare molecular characteristics of BHD-associated renal tumours and sporadic ChRCC, we conducted whole genome sequencing (WGS) of BHD-associated renal tumours and RNA-sequencing (RNA-seq) of these tumours. Using WGS data, somatic variants, tumour mutation burden (TMB), structural variants (SV), copy number alterations (CNA), mutational signature matrix and mitochondrial genome (mtDNA) alterations were investigated. In addition, we analyzed the putative origin of tumour cells and mutation acquisition timing using WGS data.

## Methods

### Patients and tumour histology

Nine unrelated patients known to be affected with BHD syndrome were evaluated in the Department of Urology and Department of Molecular Pathology, Yokohama City University, Yokohama, Japan.^16^ Each BHD patient received genetic counseling, was confirmed to have a germline *FLCN* variant, and was evaluated for clinical manifestations of BHD syndrome with a dermatologic examination, computed tomography scan (CT scan) and magnetic resonance imaging (MRI) (Table 1). Pathological diagnosis was done prior to the molecular analysis by three independent pathologists. The BHD-associated renal tumours and adjacent normal kidney specimens were frozen with liquid nitrogen and stored in −80 °C freezer until analysis. Some of the samples have already been analyzed in our previous whole exome sequencing (WES) study.^16^ Sporadic ChRCCs and sporadic oncocytomas used in the RNA-sequencing (RNA-seq) study were also surgically removed in the Department of Urology, Yokohama City University. Clinical and histological information for these samples is available in Table 2 and Supplementary Dataset 1.

**Table 1:** BHD patients and BHD-associated renal tumours analyzed in this study.

	Patient No.	Sex	Germline FLCN mutation	Mutation type	Sample No.	Age at surgery	Histology	Size (mm)	Stage
1	F42	female	c.1285dupC	Frameshift	F42-T1	49	ChRCC	30	T1a
					F42-T2	49	ChRCC	13	T1a
					F42-T3	53	ChRCC	20	T1a
2	F43	male	c.1285dupC	Frameshift	F43-T1	54	ChRCC	24	T1a
3	F59	male	c.1528_1530delGAG	Amino acid deletion	F59-T1	50	ChRCC	43	T1b
					F59-T2	50	ChRCC	14	T1a
					F59-T8	50	HOCT	32	T1a
					F59-T11	50	ChRCC	10	T1a
					F59-T12	50	ChRCC	8	T1a
4	F107	male	c.1347_1353dupCCACCCT	Frameshift	F107-T1	56	Clear cell	42	T3b
5	F123	female	c.1285dupC	Frameshift	F123-TR1	34	HOCT	27	T1a
6	F124	male	c.1285dupC	Frameshift	F124-T1	64	HOCT	26	T1a
7	F133	female	c.1285dupC	Frameshift	F133-T1	66	Unclassified	30	T1a
					F133-T2	66	ChRCC	10	T1a
8	F135	female	c.1347_1353dupCCACCCT	Frameshift	F135-T1	40	HOCT	7	T1a
9	F192	female	c.404delC	Frameshift	F192-T1	54	ChRCC	15	T1a

**Table 2:** Summary of renal tumours analyzed in this study.

	BHD-associated renal tumours	Sporadic ChRCCs	Sporadic oncocytomas
Number of patients			
Female	5	11	3
Male	4	10	4
Age (median) years of age	34-66 (50)	35-73 (61)	62-77 (69)
Histology			
ChRCC	10	21	–
HOCT	4	–	–
Clear cell RCC	1	–	–
Unclassified	1	–	–
Oncocytoma	0	–	7
Size (median) mm	7-43 (22)	10-110 (25)	11-60 (18)
Stage			
T1a	14	16	–
T1b	1	4	–
T3a	0	1	–
T3b	1	0	–

### Ethics

This study was approved by the Institutional Review Board of Yokohama City University (A200100004) and RIKEN (H20-11), and each patient has provided written informed consent for publication.

### RNA sequencing (RNA-seq)

In this study, RNA-seq of twenty-one sporadic ChRCCs and seven oncocytomas was performed (Supplementary Dataset 1). Bulk RNA-seq of sixteen BHD-associated renal tumours and five adjacent normal kidneys from ten BHD patients has been carried out in our previous study.^17^ RNAs were extracted from frozen tissues using the RNA extraction reagent ISOGEN (Nippon-gene) following the manufacturer's protocol, qualified and quantified by Bioanalyzer (Agilent Technologies). Library preparation was done using TruSeq Stranded mRNA Library Prep Kit and TruSeq RNA CD Index Kit according to the manufacturer protocol (Illumina). Sequencing was performed with HiSeq 2500 system with 126-bp reads (Illumina). Using STAR (v2.7.9a), the FASTQ files were mapped to a reference sequence generated from the GRCh37 fasta and GENCODE Release 37 comprehensive gene annotation for reference chromosomes (gencode.v19.annotation.gtf). Aligned SAM files were sorted and duplicates were marked with picard MarkDuplicates. Mapped reads were counted by featureCounts. Based on the read count data, gene expression levels were calculated as fragments per kilobase of exon per million mapped reads (fpkm). For the series of analyses from FASTQ file data to gene expression level normalization, the riboduct pipeline was used (https://github.com/msfuji/riboduct), and the details of the commands and parameter settings are available in the GitHub page. Then, these read count data and normalized data were used for further expression analysis. Gene Set Enrichment Analysis (GSEA) was done using GSEA software (v.4.2.3) (https://www.gsea-msigdb.org/gsea/index.jsp); default parameters were applied.^19^ Plots of principal component analysis (PCA) and *L1CAM* and *FOXI1* expressions were created using iDEP (integrated Differential Expression and Pathway analysis) (v.0.96) (http://bioinformatics.sdstate.edu/idep96/), a tool for RNA-seq analysis on a web browser.^20^ In the iDEP analyses, raw count data created from featureCounts was used, and default settings were used for each parameter.

### Analysis of single-cell transcriptomes

Using the single-cell RNA-seq data from our previous work, we performed gene expression analysis in BHD-associated renal tumour cells.^17^ The R package Seurat (v3.1.2) was used for the analysis.^21^ Parameter settings for clustering were resolution 2.0 for the FindClusters function, dims 1:4 for the RunUMAP function, and the package default values for the other parameters. BHD-associated HOCT cells were clustered into *L1CAM* expressing cell cluster (cL1CAM) or *FOXI1* expressing cell cluster (cFOXI1), and gene expressions in each cell cluster were investigated.

### Whole genome sequencing (WGS)

WGS was performed on sixteen BHD-associated renal tumours and nine adjacent normal kidneys from nine BHD patients (Table 1 and Supplementary Dataset 1). Genomic DNA was extracted from frozen tissues using the QIAGEN DNA Mini Kit (QIAGEN) following the manufacturer's protocol, qualified and quantified by Bioanalyzer (Agilent Technologies). The library construction was done using the TruSeq Nano DNA Library Prep Kit (Illumina) following the manufacturer's protocol. Paired-end sequencing of 150-bp reads was performed using NovaSeq6000. Reads mapping and further analyses were done using Genomon2 pipeline (v.2.6.3) (https://github.com/Genomon/genomon). Briefly, sequence reads were mapped to the reference human genome GRCh37 using BWA-mem. GATK (The Genome Analysis Toolkit) was used to realign bam files. For the Genomon2 pipeline analysis, the default settings were used for each parameter, and a control panel of normal samples was used to eliminate germline mutations and errors. We obtained the BAM file data of 49 samples of sporadic ChRCC from the data resource of The Pan-Cancer Analysis of Whole Genomes (PCAWG) study (KICH).^12^ In tumour mutation burden (TMB) analysis and mutational signature analysis, for the comparison of BHD-associated renal tumours and KICH, the output data (VCF files) using the same method described above were used. In TMB analysis, SP123975 was excluded because of the exceptionally higher number of mutations (54,708 mutations) due to DNA mismatch repair deficiency.

### Germline and somatic variant calling

Single-nucleotide variants (SNV), multi-nucleotide variants (MNV) and small insertion-deletions (Indels) were called using SAGE v2.8 (Somatic Alterations in Genome) from the Hartwig Medical Foundation (HMF: https://github.com/hartwigmedical/hmftools/releases/tag/sage-v2.8). Germline calling parameters were based on the "germline mode" in the SAGE documentation. The panel-of-normals (PON) VCFs used in the pipeline for annotation and filtering of somatic variants called by SAGE were created from 149 Japanese germline samples.^22^ Genome-wide somatic variant calls were performed with a quality cutoff of 70 and then filtered using bcftools with a quality cutoff of 70 for hotspots and 100 for other genomic locations. The maximum acceptable variant allele frequency (VAF) for the reference sample was set at 4% and the minimum VAF for the tumour was set at 2.5%. The output VCFs were filtered for PON variant sites, sites that were variant or likely fixed (MAF >0.01 and AF < 0.99; AF ≥ 0.99) in dbSNP or ALFA population samples.^22^ The resulting VCFs were annotated using ANNOVAR and SnpEff.

### Analyses of gene and structural variants (SV), and copy number alterations (CNA)

Structural Variation (SV) was called using GRIDSS 2.12.0 (the Genomic Rearrangement IDentification Software Suite)^23^ with the BAM file for each sample as the input. Scripts were used from GRIDSS- PURPLE-LINX (GPL) pipeline (v.1.3.2) (https://github.com/hartwigmedical/gridss-purple-linx). Analysis of copy number alteration (CNA) was done using CNApp (https://tools.idibaps.org/CNApp/), a tool for the quantification of copy number alterations.^24^ TUMOUR.purple.cnv.somatic.tsv, output files from the PURPLE, were used as input files. In order to minimize the copy number noise, the optimal solution for ploidy and purity in the PURPLE was selected and the noise was removed. After this process, F43-T1, F59-T11 and F59-T12 were excluded because of the high copy number noise. In the CNApp analysis, the default settings were used for each parameter.

### Mutational signature analysis

Analysis of somatic mutation signatures was performed using SigProfiler (v.3.3) (https://cancer.sanger.ac.uk/signatures/tools/) with the VCFs created from the Genomon pipeline. Somatic mutation signature profiles were decomposed to COSMIC 3.2 signatures.^25^ The default settings were used for each parameter in all of analyses.

### Putative cell-of-origin (COO) estimation

COOBoostR is a xgboost-based machine learning algorithm which aims to predict putative tissue-of-origin (TOO) or cell-of-origin (COO) (https://github.com/SWJ9385/COOBoostR).^26^ In our study, COOBoostR was applied to predict putative COO of BHD-associated renal tumours and ChRCCs. Briefly, 1-megabase window-based somatic point mutation frequencies from WGS data were calculated for individual tumour samples along with processing single-cell ATAC-seq data from normal kidney tissue^27^ by calculating 1-megabase window sequencing count density per each cell type (prespecified inside the metadata). For the parameters, default values were used except for the eta and depth (set to 0.3 and 10).

### Putative variant acquisition timing analysis

R package MutationTimeR (v.1.0.2) (https://github.com/gerstung-lab/MutationTimeR) was used to examine the timing of a second hit occurrence of the remaining *FLCN* allele in BHD-associated renal tumours.^28^ MutationTimeR is an R package used by the PCAWG consortium to calculate mutation times for 2778 whole genome sequence samples.^28^ VCFs of BHD-associated renal tumour were used and each parameter was set as default setting. A small “clusters” data.frame, which includes the estimated number of subclonal clusters, SNVs in each cluster and clonal proportions of each cluster, created from Clip pipeline (v.1.2.1) (CliP: Clonal structure identification through penalizing pairwise differences) (https://github.com/wwylab/CliP) (BioRxiv: https://doi.org/10.1101/2021.03.31.437383) was used as an input for MutationTimeR.

### Mitochondrial DNA analysis

Mitochondrial genome (mtDNA) copy number per cell in tumour and normal tissues was calculated as follows. Germline SAGE variants were extracted, the mean depth of coverage for variant sites on autosomes (mean.DPauto) or on chrM (mean.DPchrM) was calculated, and the mtDNA copy number in each tissue was estimated using the formula.^29^

This formula scales as:

$$mtCN=\frac{{mean.DP}_{chrM}}{{mean.DP}_{auto}}\left(f\times ploidy+\left(1-f\right)\times 2\right)$$

for purity (f: 0–1 for tumours, 1 for normal tissues) and ploidy (2 in normal tissues).

Also, variants in the mtDNA of BHD-associated renal tumours were analyzed using VarScan and Mutect2, from which variant unions were extracted and further filtered as described below. Filtering criteria were as follows. Position was not in (302–315, 513–525, 568–573, 956–965, 3105–3109, 5895–5899, 8270–8289, 16180–16195 (indel)). Variant reads were≥10, variant base quality score was≥20. Forward read/Total variant read ratio: >0.1 and <0.9. Mean read position: >0.15 and <0.85 (from 5′ and 3’ both). Reference read length - Variant read length was <25. Reference read mapping quality - Variant read mapping quality was <10. Reads with normal context were >80% of variant reads (except variant next to germline mutation). Variant NM - Ref NM was≤3. Remove Germline Polymorphism (germline list of SAMPLES and PCAWG (VAF >95%), dbSNP (Frequency >1%)). Calculate Noise Level in normal sample in SAMPLE and PCAWG - > remove VAF lower than noise level. Variants that passed the filter were labeled as somatic or tumour-specific dysplastic variants based on VAF differences observed in normal tissue. We analyzed mtDNA variants of sporadic ChRCCs in TCGA cohort in the same method as we stated above .^12^

### Immunohistochemistry

Immunohistochemistry was done on 4-mm-thick formalin-fixed paraffin-embedded sections of five each of *L1CAM* expressing sporadic ChRCCs (gL1CAM) and *FOXI* expressing sporadic ChRCCs (gFOXI1). In immunohistochemistry of FOXI1 protein, the tissue slides were autoclaved in Tris–EDTA buffer (pH 6.0) for antigen retrieval, then incubated with goat polyclonal FOXI1 antibody at 1:400 dilution (#ab20454; Abcam, Cambridge, United Kingdom) for 1 h at room temperature and visualized with N-Histofine Simple Stain MAX PO(G) (NICHIREI BIOSCIENCE INC., Tokyo, Japan). The FOXI1 protein was considered to be positive when nucleic and/or cytoplasmic FOXI1 staining was observed. In immunohistochemistry of L1CAM protein, the tissue slides were autoclaved in Tris–EDTA buffer (pH 9.0) for antigen retrieval, then incubated with mouse monoclonal L1CAM antibody at 1:200 dilution (clone 2C2, #ab24345; Abcam) for 1 h at room temperature and visualized with the Real EnVision Detection System, Peroxidase/DAB+, Mouse (Agilent Technologies). The L1CAM protein was considered to be positive when membranous L1CAM staining was observed.

### Statistics

Welch's two sample t-test was applied to determine whether the means of two populations were different, and differences were considered to be statistically significant at a value of *P* < 0.05. All statistical tests were 2-sided. For the analysis of the correlation between the two gene expressions, Pearson's correlation coefficient and p-value were calculated.

### Role of the funding source

The funders played no role in study design, data collection, data analyses, interpretation and writing of the report.

## Results

### BHD-associated renal tumours display totally different expression profiles from sporadic ChRCCs

**Fig. 1:**
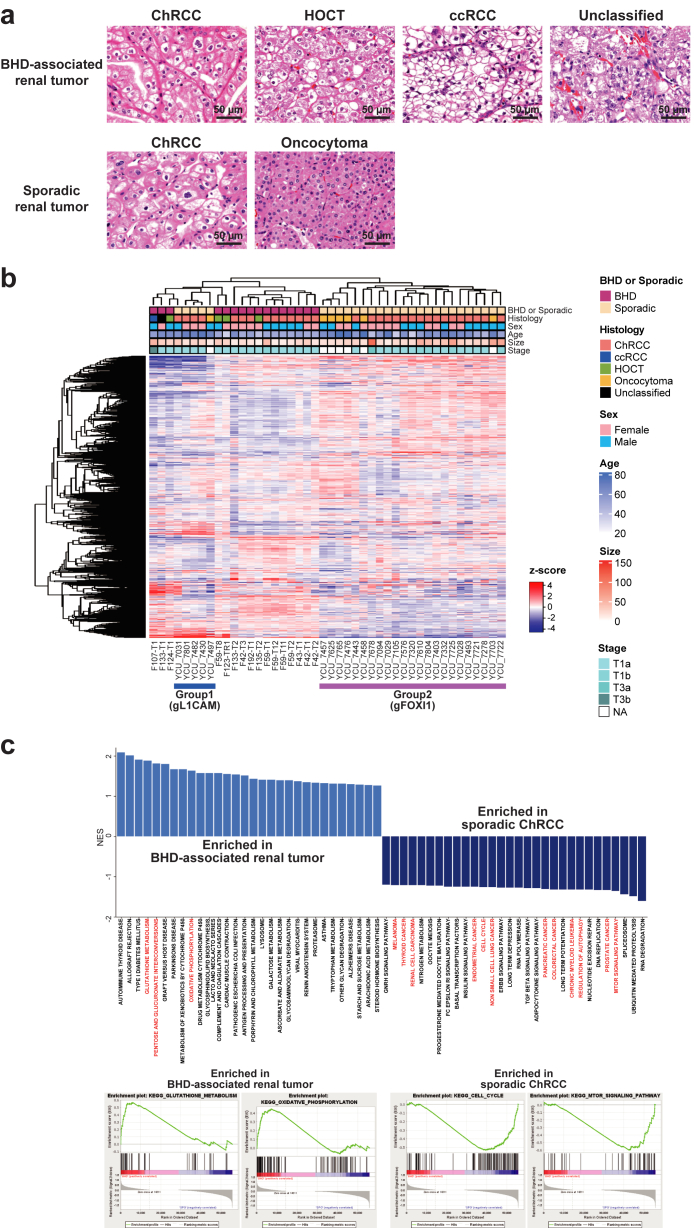
**Expression analyses highlighted unique molecular characteristics of BHD-associated renal tumours.** (a) Hematoxylin and eosin staining of renal tumours analyzed in this study. Images are shown at 400× magnification. Scale bars represent 50 μm. (b) Heatmap shows expression profiling of sixteen BHD-associated renal tumours, twenty-one sporadic ChRCC and seven sporadic oncocytomas. Sporadic ChRCC and sporadic oncocytomas was further clustered into two distinct subclusters; Group1(gFOXI1) and Group2 (gL1CAM); further analyses are done in Fig. 2 gL1CAM: group of sporadic ChRCCs with predominant expression of *L1CAM*. gFOXI1: group of sporadic ChRCCs with predominant expression of *FOXI1*. (c) Bar plot shows gene set enrichment analysis (GSEA) results comparing sixteen BHD-associated renal tumours and twenty-one sporadic ChRCCs (upper panel). Some of the GSEA enrichment plots are shown (lower panels).

We conducted RNA-seq of sixteen BHD-associated renal tumours (ten ChRCCs, four HOCTs, one clear cell renal cell carcinoma and one unclassified renal cell carcinoma) along with twenty-one sporadic ChRCCs and seven sporadic oncocytomas (Fig. 1a). A heatmap exhibited distinctive molecular characteristics in BHD-associated renal tumours compared to sporadic renal tumours (Fig. 1b). Gene set enrichment analysis (GSEA) revealed that BHD-associated renal tumours exhibited increased oxidative phosphorylation and glutathione metabolism relative to sporadic ChRCCs, whereas sporadic ChRCCs showed upregulated pathways related to various types of cancers, cell cycle and DNA replication when compared to BHD tumours, consistent with the notion that BHD-associated renal tumours might be indolent with increased mitochondrial metabolism compared to sporadic ChRCCs (Fig. 1c) (Supplementary Dataset 2).^5^^,^^30^^,^^31^

**Fig. 2:**
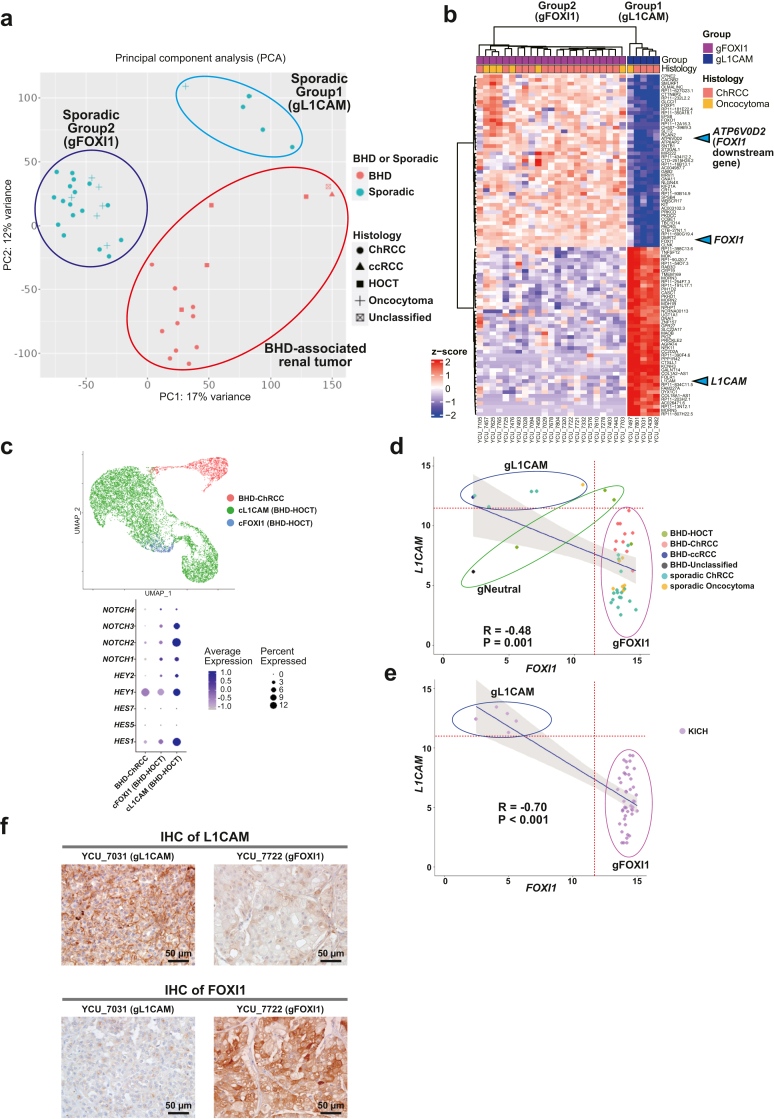
**Sporadic ChRCCs and sporadic oncocytomas formed two distinct subclusters characterized by *L1CAM* and *FOXI1* expressions**. (a) Scatter plot of principal component analysis (PCA) for Fig. 1b is shown. gL1CAM: group of sporadic ChRCCs and sporadic oncocytoma with predominant expression of *L1CAM*. gFOXI1: group of sporadic ChRCCs and sporadic oncocytomas with predominant expression of *FOXI1*. (b) Heatmap shows top fifty genes differentially expressed in Group1 (gL1CAM) and Group2 (gFOXI1). Blue triangles indicate *L1CAM, FOXI1* and its downstream gene, *ATP6V0D2*. (c) Uniform Manifold Approximation and Projection (UMAP) shows cell clusters of BHD-associated ChRCC and BHD-associated HOCT. BHD-associated HOCT is comprised of two distinct cell clusters characterized by *L1CAM* expression (cL1CAM) or by *FOXI1* expression (cFOXI1) (upper panel). Dot plot shows differentially expressing patterns of Notch genes and downstream genes in BHD-associated renal tumours (lower panel). (d) Scatter plots show an inverse correlation of *FOXI1* and *L1CAM* expression levels (upper panel). gL1CAM: group of sporadic ChRCCs with predominant expression of *L1CAM*. gFOXI1: group of sporadic ChRCCs with predominant expression of *FOXI1*. gNeutral; group of sporadic ChRCCs which were clustered into neither gL1CAM nor gFOXI1. Pearson's correlation coefficient and p-value are shown. (e) Scatter plot shows an inverse correlation of *FOXI1* and *L1CAM* expressions (lower panel). Pearson's correlation coefficient and p-value are shown. (f) Immunohistochemistry (IHC) of L1CAM protein (upper panel) or FOXI1 protein (lower panel) in sporadic ChRCCs. Scale bars represent 50 μm.

Importantly, sporadic ChRCCs and sporadic oncocytomas were further clustered into two distinct clusters, and differentially expressed genes (DEGs) analysis identified *L1CAM, FOXI1* and its downstream gene, *ATP6V0D2* as molecular markers for these distinct clusters (Group1 and 2) (Fig. 1, Fig. 2b, Fig. 1, Fig. 2a and Fig. 1, Fig. 2b) (Supplementary Dataset 3). In renal development, Notch signalling regulates a differentiation of ureteric bud either into *L1CAM*-positive principal cells or into *FOXI1*-positive intercalated cells.^18^^,^^16^ Of note, our single-cell transcriptome analysis has uncovered transcriptomic intra-tumour heterogeneity in BHD-associated HOCT, comprised of two distinct cell clusters of *L1CAM* expressing cells and *FOXI1* expressing cells in which expression patterns of Notch signalling-associated genes were different, suggesting that expression patterns of *L1CAM* and *FOXI1* determined by Notch signalling may associate with diverse molecular characteristics of renal tumours (Fig. 2c) (Supplementary Fig. S1a).^17^ In addition, we observed opposing correlation between *L1CAM* expressions and *FOXI1* expressions both in our cohort and in the TCGA ChRCC cohort (KICH), further suggesting that mutually exclusive expressions of *L1CAM* and *FOXI1* may confer intra-tumour and inter-tumour heterogeneity on BHD-associated HOCT cells and sporadic renal tumours, respectively (Fig. 2d, e and f) (Supplementary Fig. S1b).

### BHD-associated renal tumours most commonly harbour biallelic *FLCN* alterations but fewer mutations and copy number alterations

**Fig. 3:**
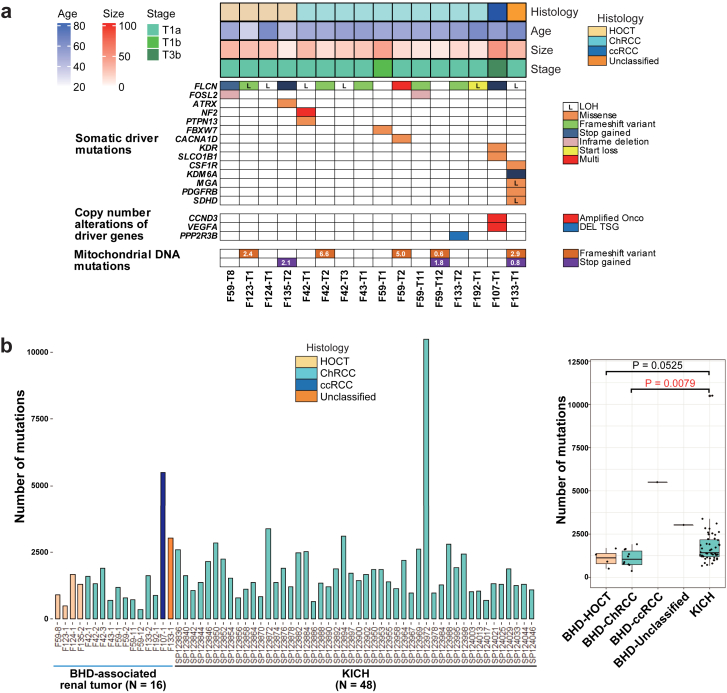
**BHD-associated renal tumours harbour less numbers of gene variants compared to sporadic ChRCCs**. (a) Mutation matrices show histology, clinical characteristics (age, size and stage) and somatic variants revealed by WGS of sixteen BHD-associated renal tumours. (b) Bar plots (left panel) and box plots (right panel) show the number of mutations in sixteen BHD-associated renal tumours and forty-eight sporadic ChRCCs in TCGA cohort (KICH). Welch's two sample t-test was used.

We conducted WGS analysis of sixteen BHD-associated renal tumours and nine adjacent normal kidneys from nine BHD patients (Table 1 and Supplementary Dataset 1). WGS analysis in this study, together with our previous WES analysis, confirmed somatic second hit *FLCN* alterations in all of the BHD-associated renal tumours (Fig. 3a) (Supplementary Dataset 4).^16^ In addition, WGS analysis of BHD-associated renal tumours demonstrated that very few genes were commonly mutated except for *FLCN,* as was observed in our previous WES study, suggesting that *FLCN* alteration should be the main driver for BHD-associated renal tumourigenesis.^16^ In F107 case, interestingly, amplification of *VEGFA* and *CCND3* on chromosome 6 was observed, which may lead to the formation of clear cell renal cell carcinoma. F133-T1, whose histology was unclassified renal cell carcinoma, harboured a variant in *succinate dehydrogenase D (SDHD)*, a gene responsible for *SDH*-associated tumour predisposition syndrome.^32^^,^^33^

The average number of mutations per tumour was 1107, 1090 and 1650 in BHD-associated ChRCCs, BHD-associated HOCTs and sporadic ChRCCs in the TCGA cohort (KICH), respectively, supporting that BHD-associated renal tumours have less inter-tumour heterogeneity than sporadic ChRCCs (Fig. 3b, and Supplementary Dataset 5). In fact, BHD-associated renal tumours did not show increased number of mutations in an age-dependent manner, whereas sporadic ChRCCs in the TCGA cohort did exhibit an age-dependent increase in number of mutations, further supporting the notion that BHD-associated renal tumours have less inter-tumour heterogeneity compared to ChRCCs (Supplementary Fig. S2a).

Of note, we observed no common structural variants in BHD-associated renal tumours, suggesting that structural variants may not be key drivers for BHD-associated renal tumourigenesis (Supplementary Fig. S2b). High-resolution copy number alteration (CNA) analysis using WGS data exhibited that CNAs of BHD-associated renal tumours were considerably different from those already reported in sporadic cases; fewer CNAs were observed in BHD-associated HOCT and BHD-associated ChRCC compared to those in BHD-associated clear cell renal cell carcinoma, which might reflect the indolent nature of BHD-associated HOCT and BHD-associated ChRCC. Loss of chromosomes 1, 2, 6, 10, 13 and 17, which is characteristic of sporadic ChRCC, was not observed in BHD-associated renal tumours (Supplementary Fig. S2c).^1^^,^^12^^,^^16^^,^^31^

### BHD-associated renal tumours demonstrate unique mutational signatures

Mutational signatures are imprints of DNA damage and repair processes that have been operative during tumourigenesis and can provide insights into environmental and endogenous causes of cancer and biological implications of somatic mutations.^25^^,^^34^ In this study, mutational signature analysis was performed using WGS data from 16 BHD-associated renal tumours and 49 sporadic ChRCCs in TCGA cohort (Supplementary Fig. S3a). BHD-associated renal tumours and sporadic ChRCCs in the TCGA cohort generally demonstrated similar patterns of mutational signatures. However, some of the signatures were different between these tumours, including SBS1 (Spontaneous deamination of 5-methylcytosine, clock-like signature), SBS2 (APOBEC activity), SBS5 (Unknown, clock-like signature), SBS13 (APOBEC activity), insertions and deletions (ID)2 (Slippage during DNA replication of the template DNA strand) and ID8 (DBS repair by non-homologous end-joining), highlighting molecular differences of these tumours.

### BHD-associated renal tumours harbour mtDNA with higher copy numbers and fewer variants

**Fig. 4:**
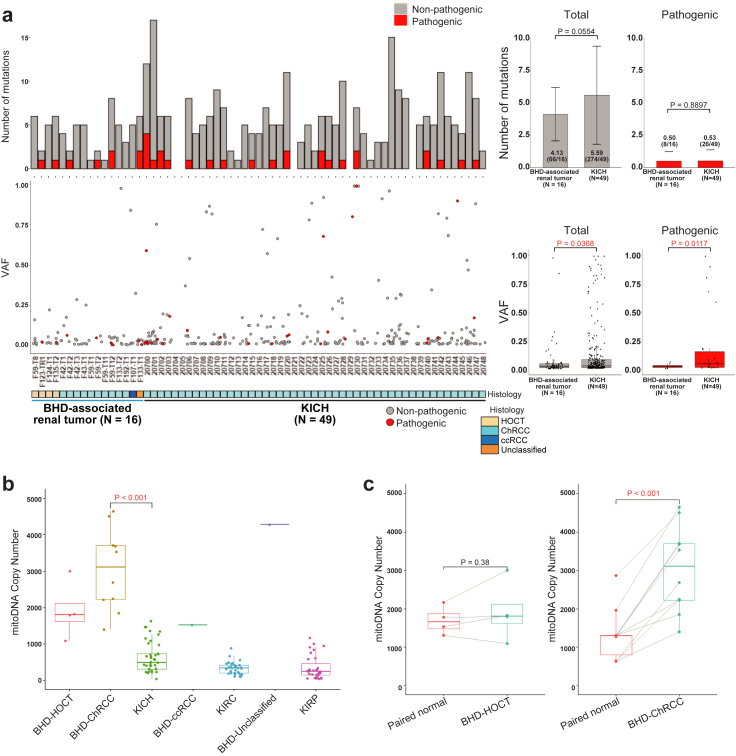
**BHD-associated renal tumours harbour higher mtDNA copy number with fewer variants compared to sporadic ChRCCs**. (a) Bar plots of number of mutations (left upper panel) and scatter plots of variant allele frequencies (VAF) (left lower panel) in mtDNA from sixteen BHD-associated renal tumours and forty-nine sporadic ChRCCs in TCGA cohort (KICH) are shown. Bar graphs show average number of variants shown in left upper panel (right upper panel). Box plots show average VAF shown in left lower panel (right lower panel). Welch's two sample t-test was used. (b) Box plot shows the mtDNA copy number in BHD-associated renal tumours and various sporadic renal tumours. BHD-Clear cell: BHD-associated clear cell renal cell carcinoma. BHD-Unclassified: BHD-associated unclassified renal cell carcinoma. KICH: sporadic ChRCCs in TCGA cohort. KIRC: sporadic clear cell renal cell carcinomas in TCGA cohort. KIRP: sporadic papillary renal cell carcinomas in TCGA cohort. Welch's two sample t-test was used. (c) Box plots show mitochondrial DNA copy number in each histological type of BHD-associated renal tumours and their adjacent normal kidneys (Paired normal). Welch's two sample t-test was used.

A notable proportion (22.2%) of sporadic ChRCCs harbor non-silent mtDNA variants without any variants in nuclear-encoded kidney cancer-associated genes, whereas BHD-associated renal tumours harbor no disruptive alterations in mtDNA, suggesting that non-silent mtDNA variants in sporadic ChRCCs may be drivers of tumourigenesis of sporadic ChRCCs whereas mitochondrial function is most likely retained in BHD-associated renal tumours.^12^^,^^29^^,^^35^ Consistent with these results, heteroplasmic level or variant allele frequencies (VAFs) of total and pathogenic variants in mtDNA of BHD-associated renal tumours were significantly lower than those of sporadic ChRCCs (*p* = 0.0368 and 0.0117, respectively) (Fig. 4a) (Supplementary Dataset 6). mtDNA copy numbers were higher in BHD-associated renal tumours compared to sporadic ChRCCs and variable depending on each histology (Fig. 4b and c) (Supplementary Fig. S4).

### BHD-associated renal tumours and sporadic ChRCC may arise from different normal nephron cells

**Fig. 5:**
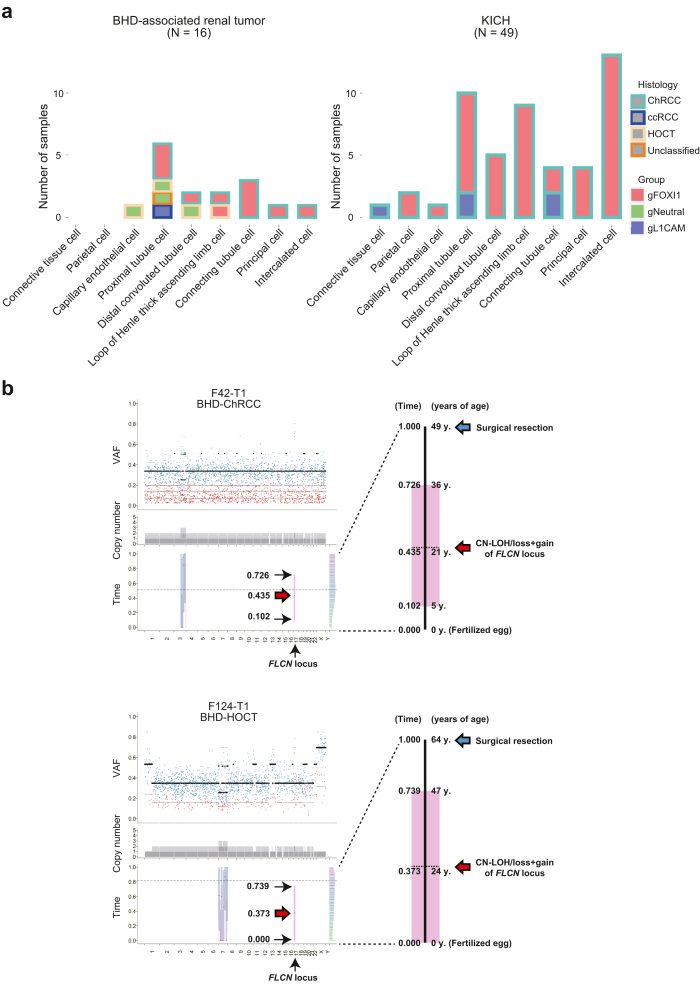
**Bioinformatic algorithms depict natural history of BHD-associated renal tumourigenesis**. (a) The bar plots show putative cell of origin of BHD-associated renal tumours (left panel) and sporadic ChRCCs in TCGA cohort (KICH) (right panel) predicted by COOBoost algorithm. Groups of gFOXI1, gNeutral and gL1CAM correspond to those in Fig. 2d and e. (b) The plots show the results of MutationTimeR algorithm. The dot plots show variant allele frequencies (VAF) of point mutations; purple dot indicates clonal mutation occurred at late stage, green dot indicates clonal mutation occurred at early stage, blue dot indicates clonal mutation occurred at unknown stage, red dot indicates subclonal mutation (left upper panels in each case). The stacked barplots show copy number of each allele; dark and light grey bars indicate major and minor allele, respectively (left middle panels in each case). The box plots with median values and 95% confidence intervals indicate predicted mutation timings of primary and secondary gains; blue bar indicates mono-allelic gains (N:1), pink bar indicates copy neutral (CN)-LOH/loss + gain (N:0) and green bar indicates bi-allelic gains (N:2) (left lower panels in each case). The histograms show magnified timelines of left lower panels in each case (right panels in each case).

A recent machine learning algorithm integrating bulk or single cell-level ATAC-seq (Assay for Transposase-Accessible Chromatin using sequencing) data of normal cell types with somatic point mutation frequency derived from the WGS data of tumours enables us to predict putative cancer cell-of-origin (COO), based on a hypothesis that the somatic variants might preferentially accumulate outside of the open chromatin regions of the cancer cell of origin.^36^ In this study, we applied the COOBoostR algorithm to predict a putative cancer COO of BHD-associated renal tumours and sporadic ChRCCs.^26^ Interestingly, the COOBoostR predicted that BHD-associated renal tumours and sporadic ChRCCs may arise from a variety of normal nephron cells. The most frequently predicted cell-of-origin was proximal tubules for BHD-associated renal tumours and intercalated cells for sporadic ChRCCs, implying the possibility that these two tumour types might arise from different cells of origin (Fig. 5a) (Supplementary Dataset 7). Of note, none of *L1CAM* expressing tumours in BHD-associated renal tumours and sporadic ChRCCs might arise from intercalated cells, suggesting that cell-of-origin may determine tumour characteristics of these tumours.

### Second hit mutations of the *FLCN* gene may occur in early third decade of life in BHD patients

Recent bioinformatics methodology allows us to predict the timing of the occurrence of a large duplication from the proportion of point variants in that duplicated region.^28^ We applied the MutationTimeR pipeline to our WGS data and predicted variant acquisition timing. In our WGS study, two samples of F42-T1 (BHD-associated ChRCC) and F124-T1 (BHD-associated HOCT) harboured a 17p copy neutral loss of heterozygosity (CN-LOH)/loss + gain, where *FLCN* gene was located. Therefore, we applied the MutationTimeR pipeline to these cases and found that a second hit mutation in the remaining *FLCN* gene may have occurred at median age of 21 years (ranging from 5 to 36 years of age) in F42-T1 patient and median age of 24 years (ranging from 0 to 47 years of age) in F124-T1 patient (Fig. 5b) (Supplementary Fig. S5).

## Discussion

Here, we present comparative analyses of BHD-associated renal tumours and sporadic renal tumours, which exhibited molecular similarities as well as differences between these histologically similar renal tumours (Table 3). WGS analysis revealed that very few genes were commonly mutated in BHD-associated renal tumours except for *FLCN* and no common structural variants were observed. Therefore, *FLCN* alteration appears to be a relatively strong tumour driver given the fact that *FLCN*-deficiency leads to kidney cell proliferation in both *in-vivo* and *in-vitro* models, whereas mutation of other kidney cancer-associated genes including *VHL* and fumarate hydratase *(FH)* in genetically manipulated murine models only develop renal cysts.^6^^,^^9^^,^^37^, ^38^, ^39^, ^40^ Taken together, BHD-associated renal tumourigenesis may be largely attributed to the exclusive loss of *FLCN* as the main tumour driver.

**Table 3:** Molecular differences between BHD-associated renal tumours and sporadic ChRCCs.

	BHD-associated renal tumours	Sporadic ChRCCs
Expressions		
Upregulated pathways (GSEA)	Oxydative phosphorylation, Glutathione metabolism	Cancer-related pathways, Cell cycle, DNA replication
Heterogeneity	Intra-tumour heterogeneity conffered by mutually exclusive expressions of *L1CAM* and *FOXI1*	Inter-tumour heterogeneity conffered by mutually exclusive expressions of *L1CAM* and *FOXI1*
Nuclear DNA		
Driver gene mutation	*FLCN*	*TP53, PTEN*
Copy number alteration (CNA)	Low	Copy loss of chr 1, 2, 6, 10, 13 and 17
Tumour mutation burden (TMB)	Low	High
Mutational signature	SBS1, SBS5, SBS40	SBS1, SBS5, SBS40
Cell of origin (most frequent)	Proximal tubule cell	Intercalated cell of collecting duct
Timing of second hit alteration	Early twenties	–
Mitochondrial DNA		
Variant allele frequency (VAF)	Low	High
Copy number	High	Low

In this study, we identified inter-tumour heterogeneity of sporadic ChRCCs, characterized by mutually exclusive expressions of *L1CAM* and *FOXI1*. *FOXI1* is an important transcription factor for differentiation of intercalated cells of the collecting duct and, importantly, Notch signalling regulates differentiation of ureteric bud cells either into *L1CAM*-positive principal cells of the collecting duct when Notch signalling is active, or into *FOXI1*-positive intercalated cells of the collecting duct when Notch signalling is inactive.^18^ Notably, defective Notch signalling drives renal cyst formation and yields intratumour heterogeneity.^41^^,^^42^ Our single-cell transcriptomic analysis of BHD-associated HOCT tumours revealed that expressions of *Notch* genes as well as those of *Notch* downstream genes including *HES* and *HEY* family members were increased in the cluster of *L1CAM* expressing cells, suggesting that Notch signalling may associate with transcriptomic intratumour heterogeneity (tITH) of BHD-associated renal tumours. However, in sporadic ChRCCs, although expressions of genes in the Notch signalling-mediated network, *i.e., ARNT*, *PARP1*, *RB1* and *STAT3*, exhibited positive correlation with *L1CAM* expression and inverse correlation with *FOXI1* expression, expressions of *HES1, HES5, HES7, HEY1* and *HEY2*, direct downstream genes of Notch signalling, were not correlated with *L1CAM* or *FOXI1* expression, suggesting that Notch signalling may be dysregulated in sporadic ChRCCs (Supplementary Fig. S6).

Recent bioinformatics methodology allows us to predict the putative timing of a large duplication from the proportion of point variants in that duplicated region.^28^ According to this methodology, chromosome 3p loss with concurrent chromosome 5q gain may occur during childhood to adolescence and the remaining *VHL* allele may be lost after this event in sporadic clear cell renal cell carcinoma.^43^ On the other hand, chromosome 3p loss with concurrent chromosome 5q gain may occur during childhood to adolescence in VHL-associated kidney cancer, suggesting that the earlier biallelic loss of the *VHL* gene may result in earlier development of kidney cancer compared to sporadic clear cell renal cell carcinoma.^43^ Our timing prediction analysis has shown that second hit alterations of *FLCN* may occur during the BHD patients’ early twenties, further supporting the concept that the earlier biallelic loss of causative genes in inherited renal tumours may lead to earlier tumour development compared to sporadic renal tumour.

So far, we have experienced only one BHD-associated oncocytoma and hence, the frequency of oncocytoma in BHD-associated renal tumours in our cohort is relatively low compared to that in other cohorts.^16^^,^^44^ Although a genotype–phenotype correlation has not been investigated in BHD syndrome, it is urgent to interrogate the elements that possibly affect histological variations across the cohorts.

The findings of this study, which delineate the molecular similarities as well as differences between BHD-associated renal tumours and sporadic renal tumours, further our understanding of the tumourigenesis of these renal tumours. These findings provide a foundation for the management of these tumours as well as for the development of targeted therapeutics approaches for BHD-associated tumours and sporadic renal tumours.

## Contributors

Hisashi Hasumi had full access to all the data in the study and takes responsibility for the integrity of the data and the accuracy of the data analysis. Study concept and design: Jikuya, Schmidt, Linehan, Yao, Nakagawa, Hasumi. Acquisition of samples: Jikuya, Tatenuma, Komeya, H. Ito, Y. Ito, Muraoka, Hamanoue, Makiyama, Hasumi. Acquisition of data: Jikuya, Maejima, An, Ju, Kim, Imoto, Nakagawa. Analysis and interpretation of data: Jikuya, Johnson, An, Ju, Lee, Ha, Song, Kim, Okawa, Sasagawa, Kanazashi, Fujita, Imoto, Mitome, Ohtake, Noguchi, Kawaura, Iribe, Aomori, Furuya, Kato, Fujii, Tamura, Baba, Suda, Kodama, Shuch, Nakagawa, Hasumi. Drafting of the manuscript: Jikuya, Ricketts, Schmidt, Linehan, Nakagawa, Hasumi. Critical revision of the manuscript for important intellectual content: All authors. Statistical analysis: Jikuya, Johnson, Maejima, An, Ju, Lee. Obtaining funding: Komeya, H. Ito, Y. Ito, Makiyama, Yao, Nakagawa, Hasumi. Administrative, technical, or material support: Jikuya, Hasumi. Supervision: Nakagawa, Hasumi. Jikuya and Hasumi have verified the underlying data. All authors read and approved the final version of the manuscript.

## Data sharing statement

All of sequencing data were deposited in National Bioscience Database Center (NBDC) under the accession number JGAS000564, https://humandbs.biosciencedbc.jp/en/hum0277-v2.

## Declaration of interests

The authors declare no competing interests.

## Appendix A Supplementary data

Supplementary Fig. S7

Caption for supplementary material
