# Supplementary figures and images for: Comparative analyses define differences between BHD-associated renal tumour and sporadic chromophobe renal cell carcinoma
Source: eBioMedicine. 2023 May 12;92:104596. doi: 10.1016/j.ebiom.2023.104596 (PMC10200853; doi:10.1016/j.ebiom.2023.104596)

Supplementary Figure 1

a

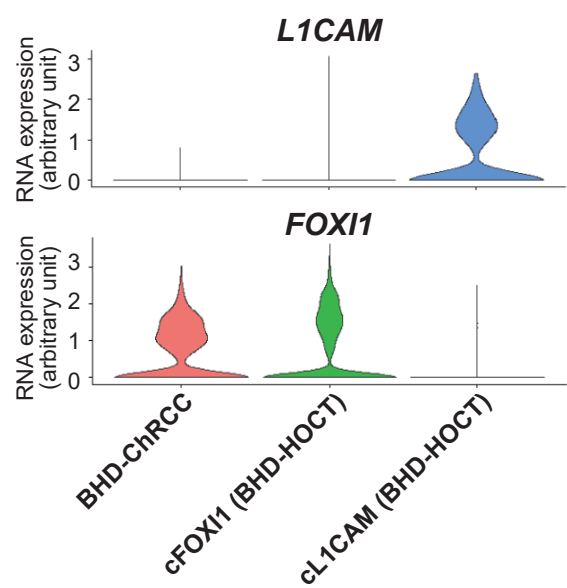

b

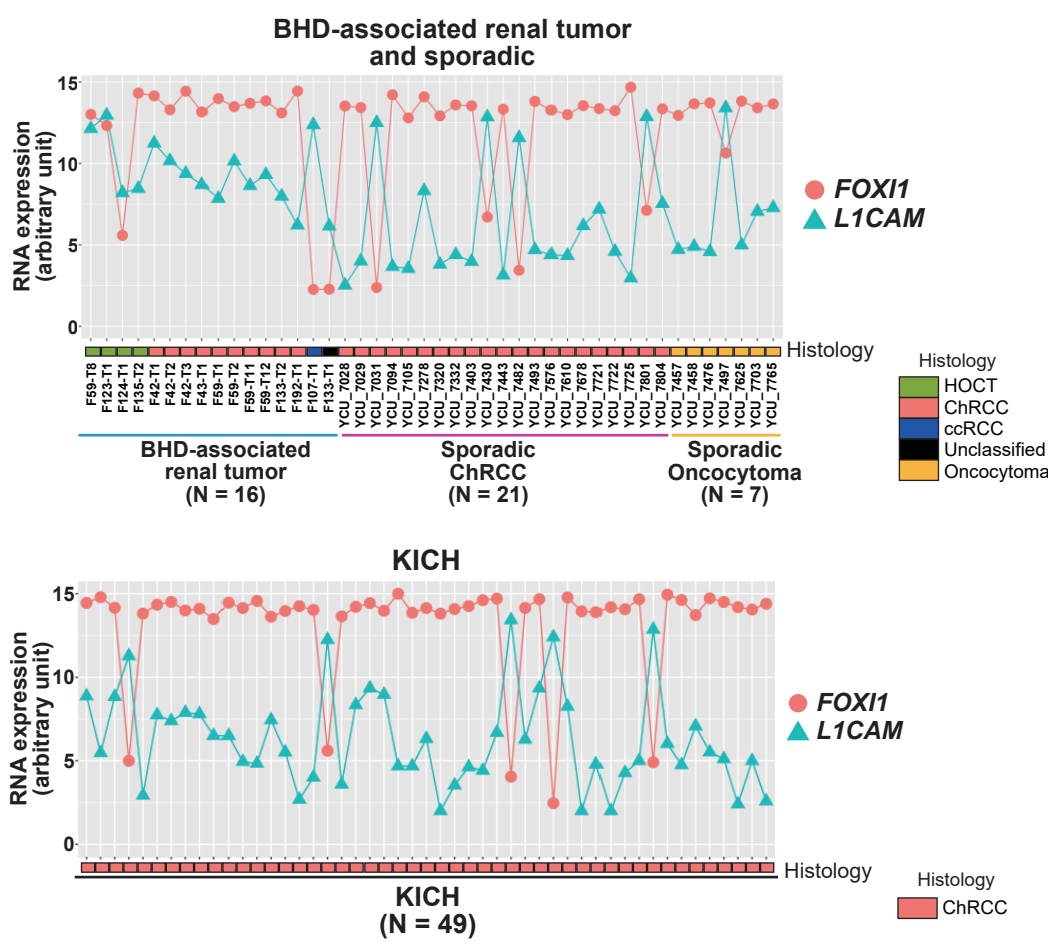

Supplement: Supplementary Fig. S1 [file mmc8.pdf]

Supplementary Figure 2

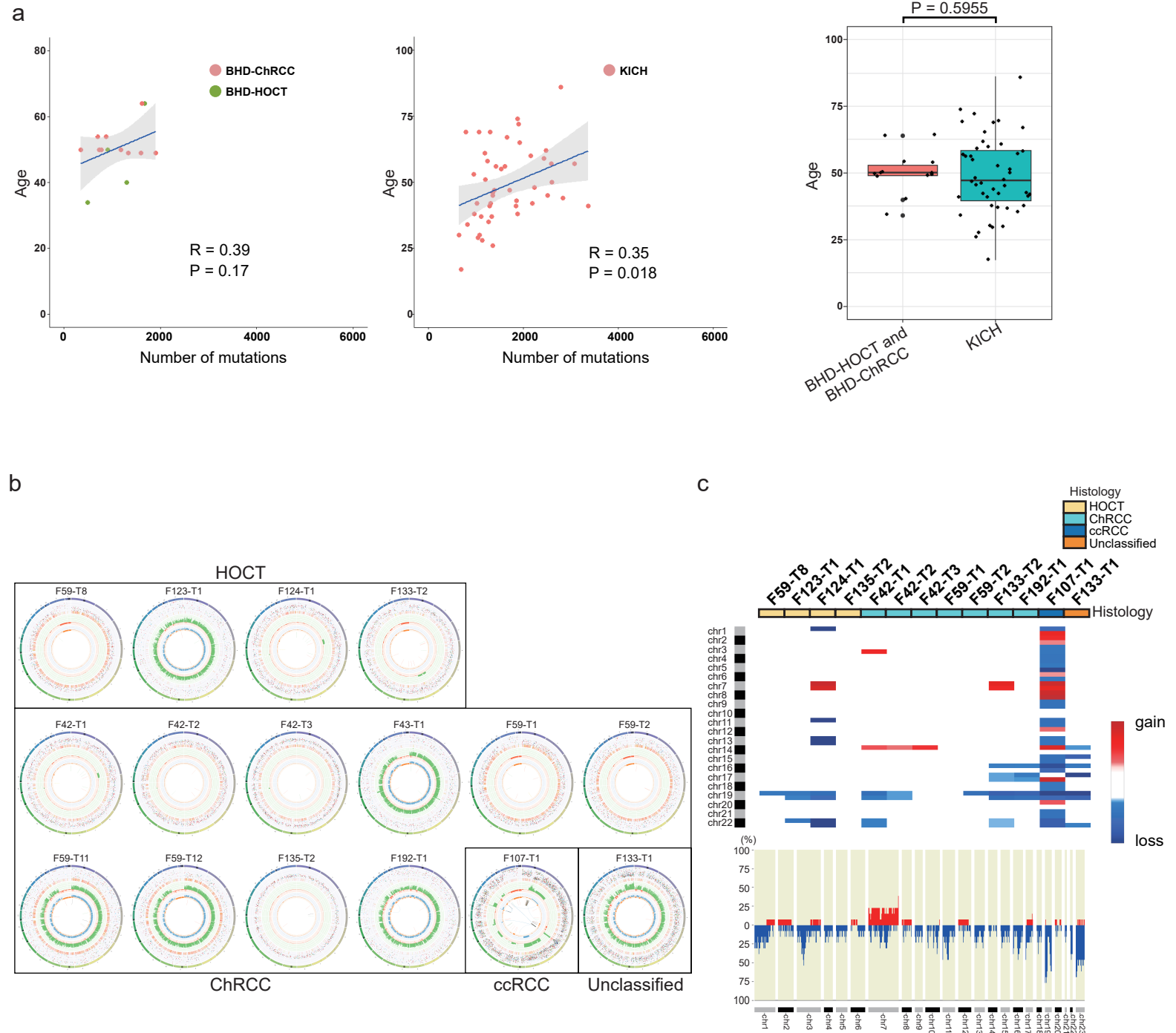

Supplement: Supplementary Fig. S3 [file mmc9.pdf]

Supplementary Figure 3

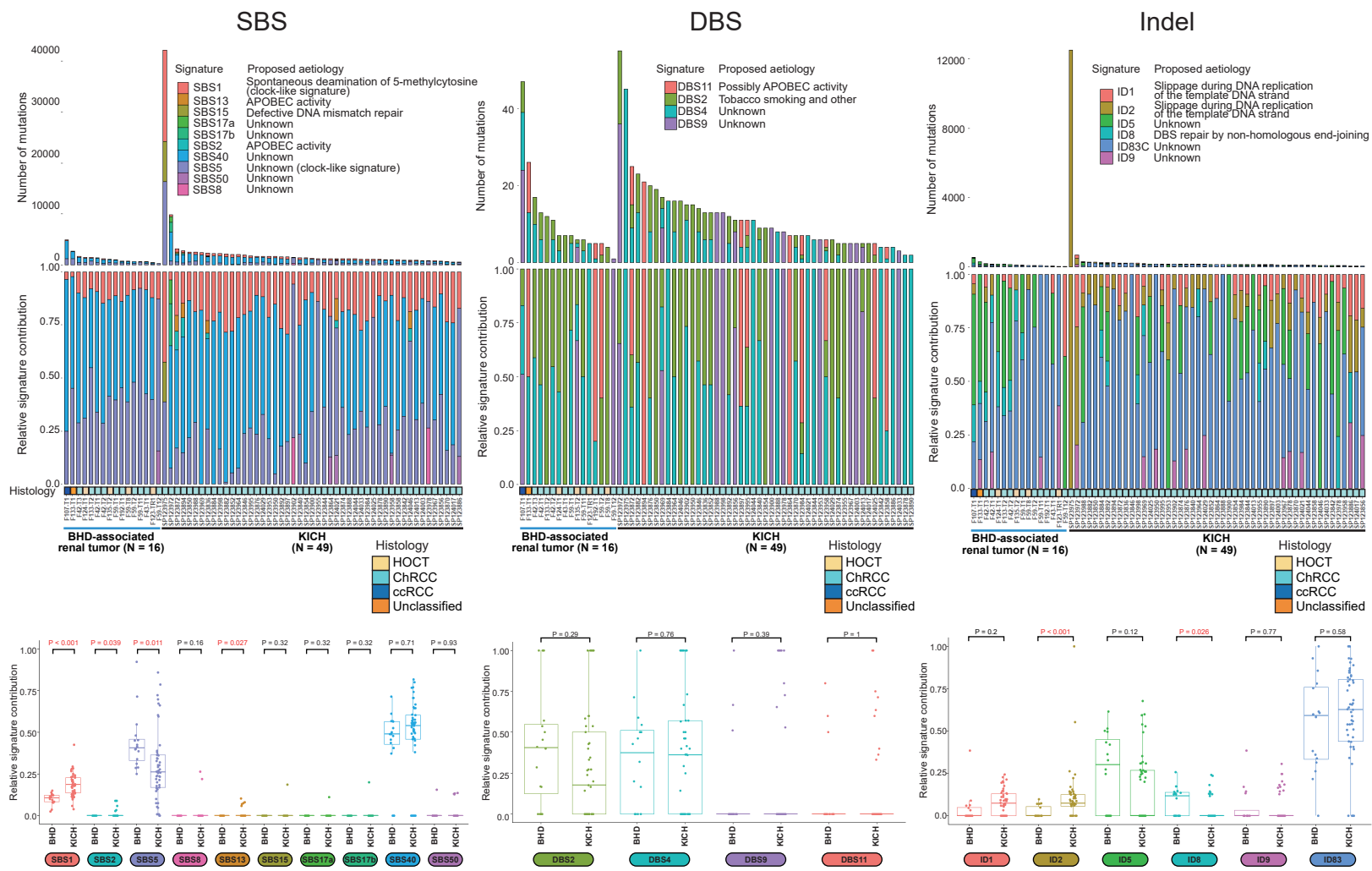

Supplement: Supplementary Fig. S4 [file mmc10.pdf]

Supplementary Figure 4

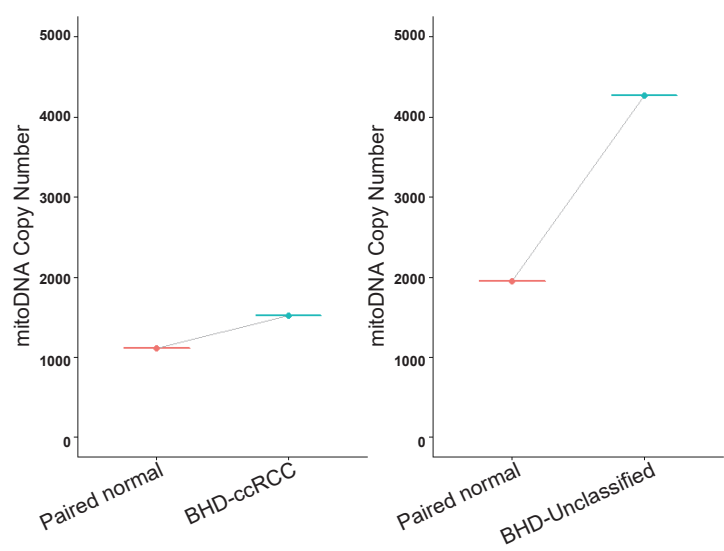

Supplement: Supplementary Fig. S5 [file mmc11.pdf]

Supplementary Figure 6

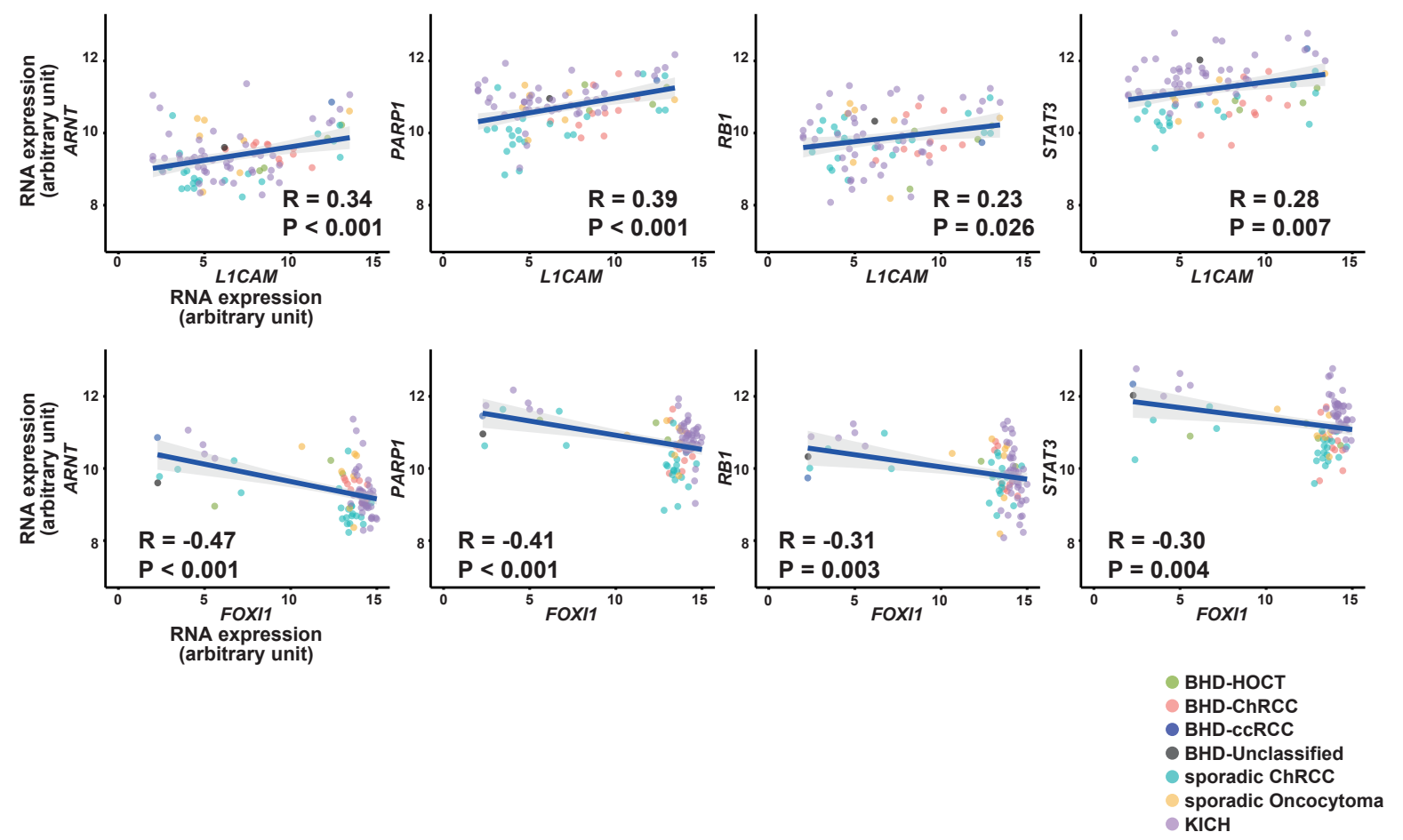

Supplement: Supplementary Fig. S7 [file mmc13.pdf]
