# Supplementary material for: Comparative analyses define differences between BHD-associated renal tumour and sporadic chromophobe renal cell carcinoma
Source: eBioMedicine. 2023 May 12;92:104596. doi: 10.1016/j.ebiom.2023.104596 (PMC10200853; doi:10.1016/j.ebiom.2023.104596)
Supplement: Supplementary Fig. S6 [file mmc12.pdf]

Supplementary Figure 5

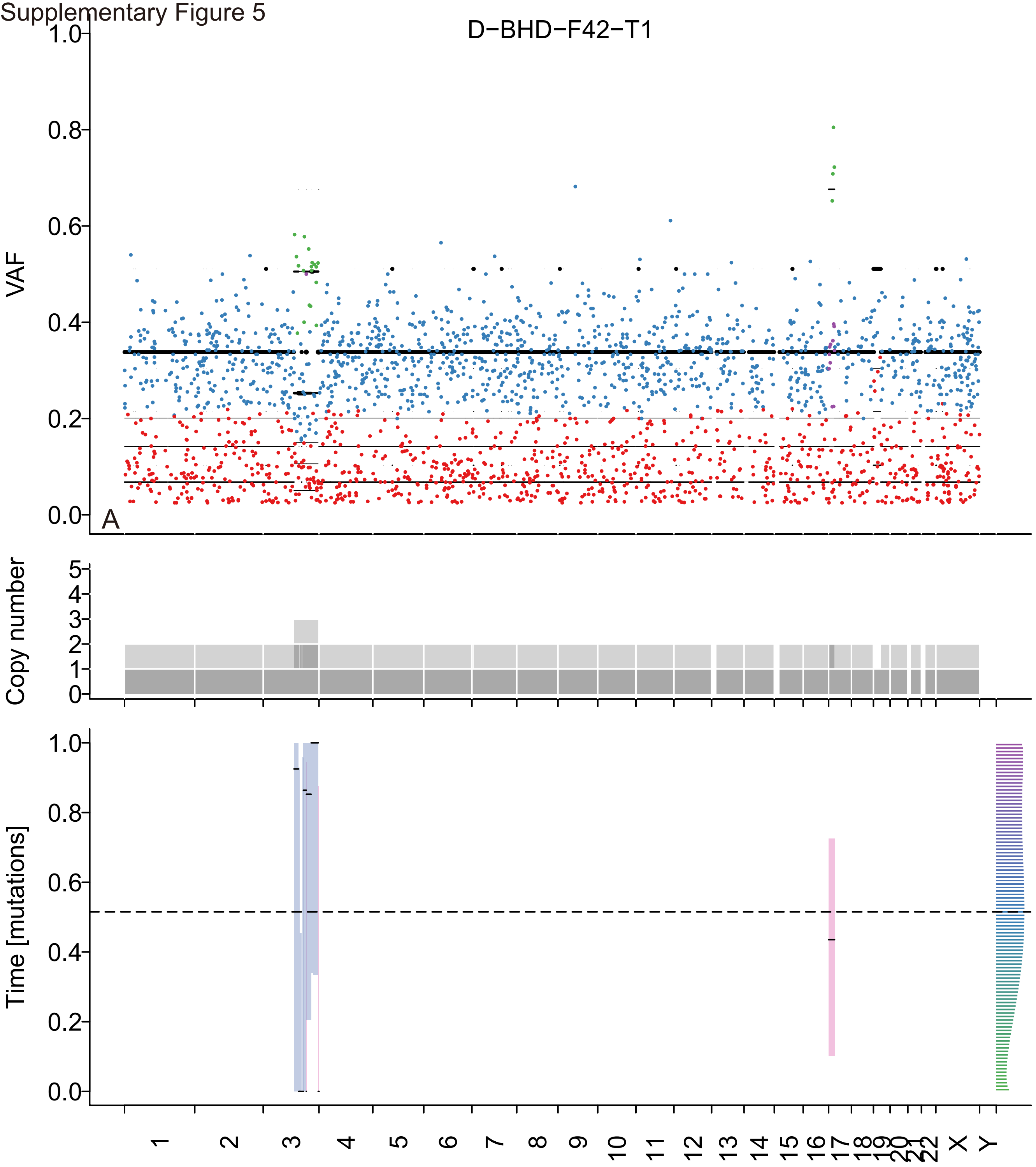

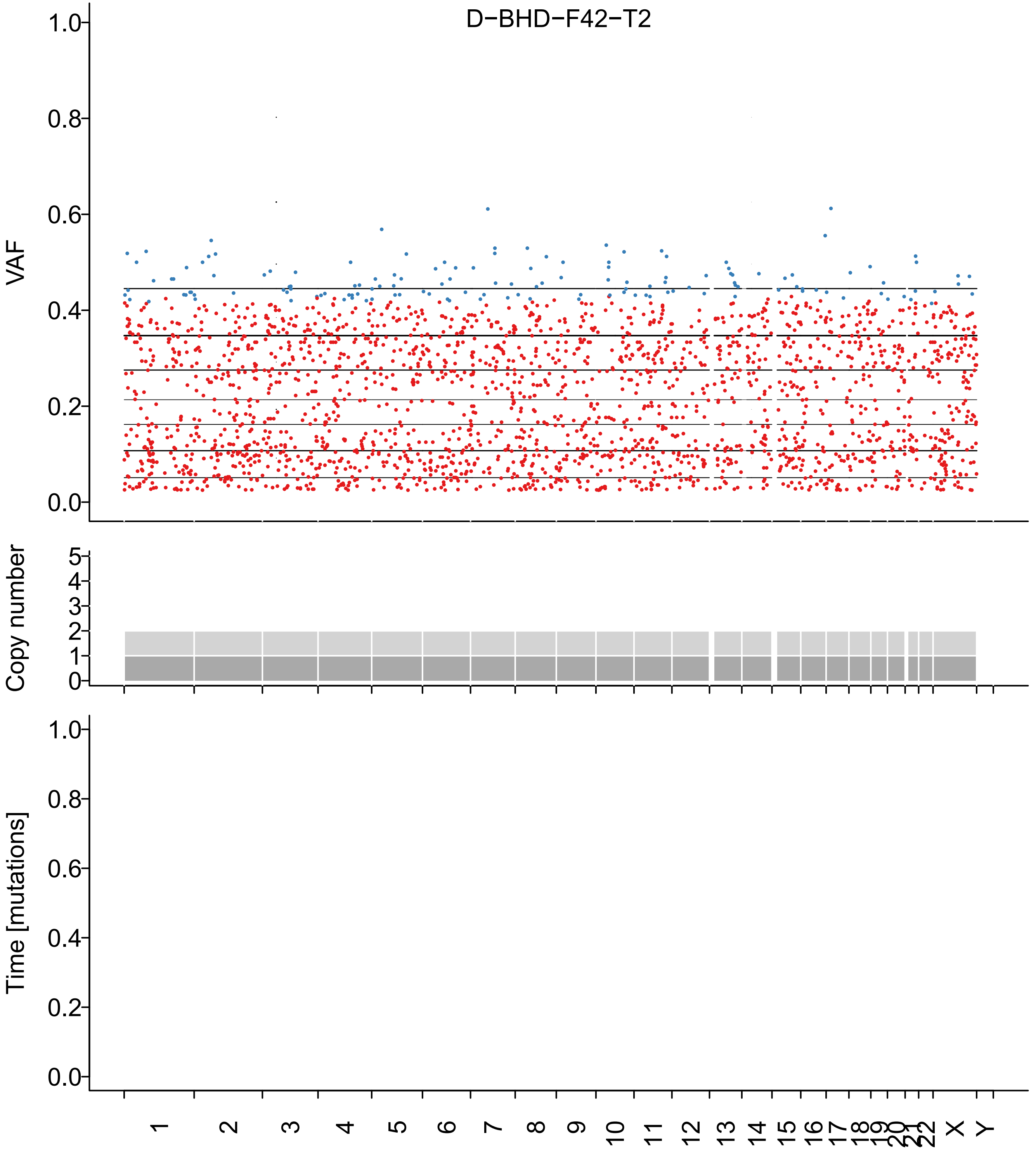
D−BHD−F42−T2
VAF
1.0
0.8
0.6
0.4
0.2
0.0
Copy number
5
4
3
2
1
0
Time [mutations]
1.0
0.8
0.6
0.4
0.2
0.0
1
2
3
4
5
6
7
8
9
10
11
12
13
14
15
16
17
18
19
20
21
22
X
Y

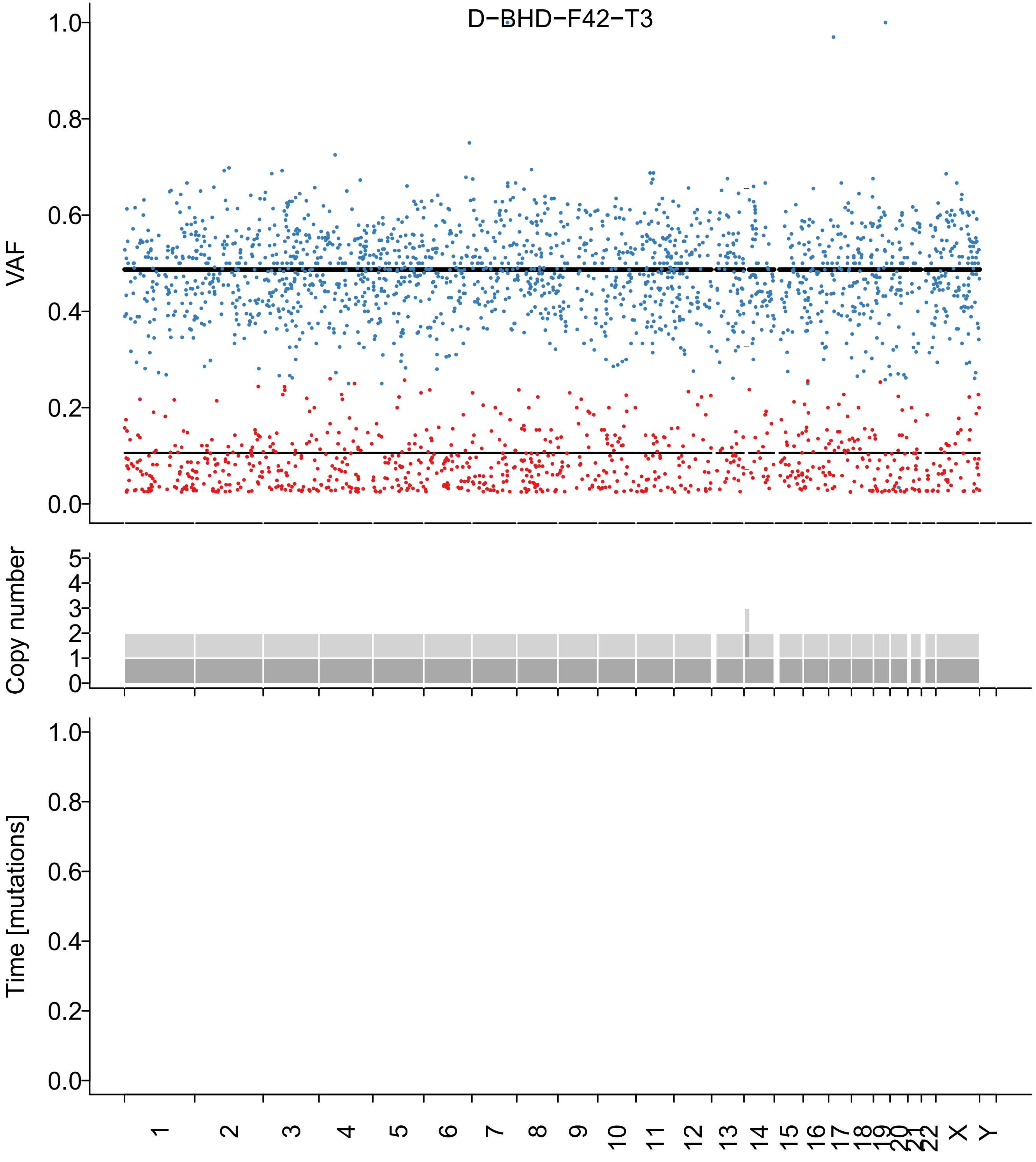

D−BHD−F42−T3
VAF
Copy number
Time [mutations]
1
2
3
4
5
6
7
8
9
10
11
12
13
14
15
16
17
18
19
20
21
22
X
Y

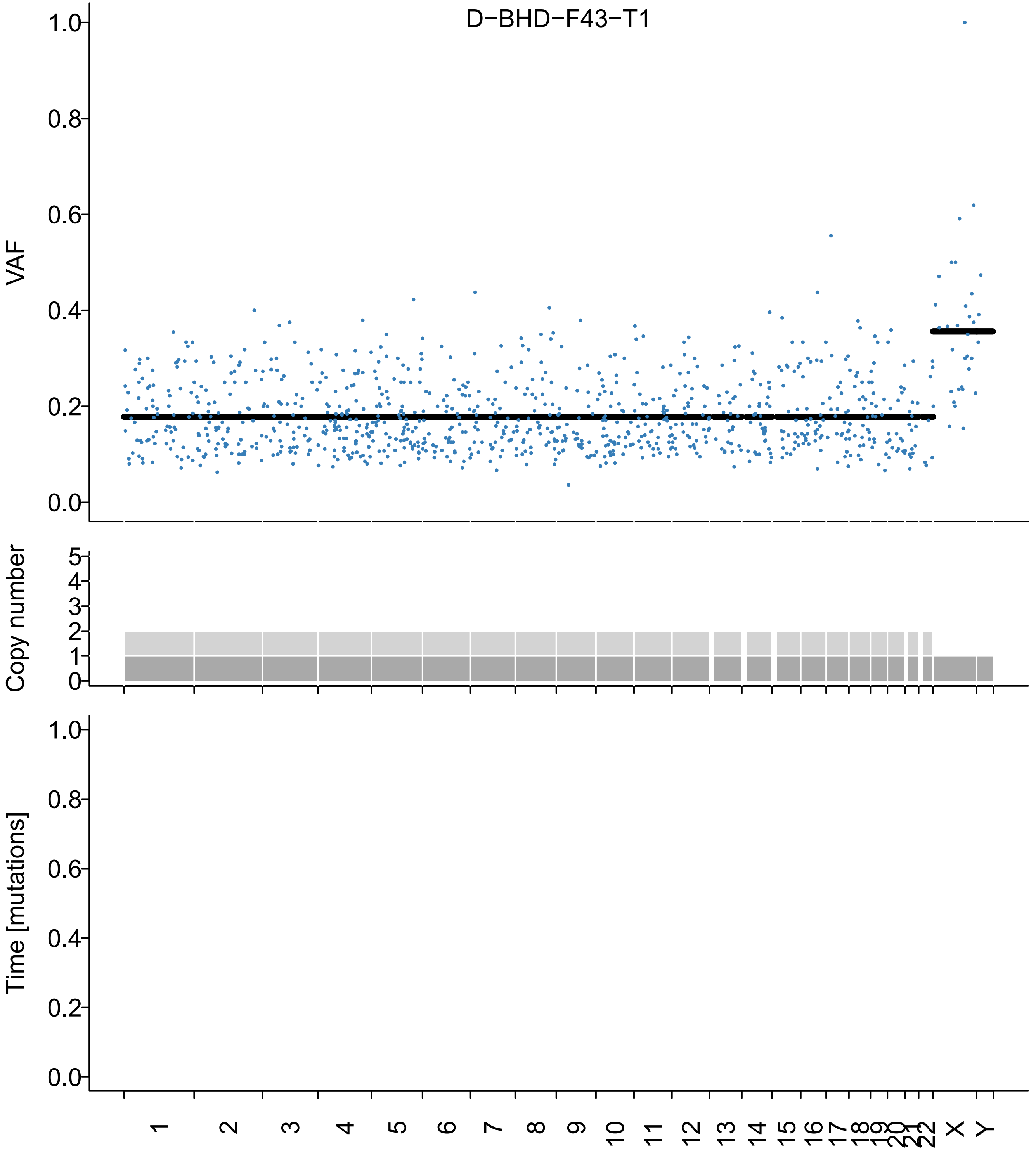
D−BHD−F43−T1
VAF
1.0
0.8
0.6
0.4
0.2
0.0
Copy number
5
4
3
2
1
0
Time [mutations]
1.0
0.8
0.6
0.4
0.2
0.0
1
2
3
4
5
6
7
8
9
10
11
12
13
14
15
16
17
18
19
20
21
22
X
Y

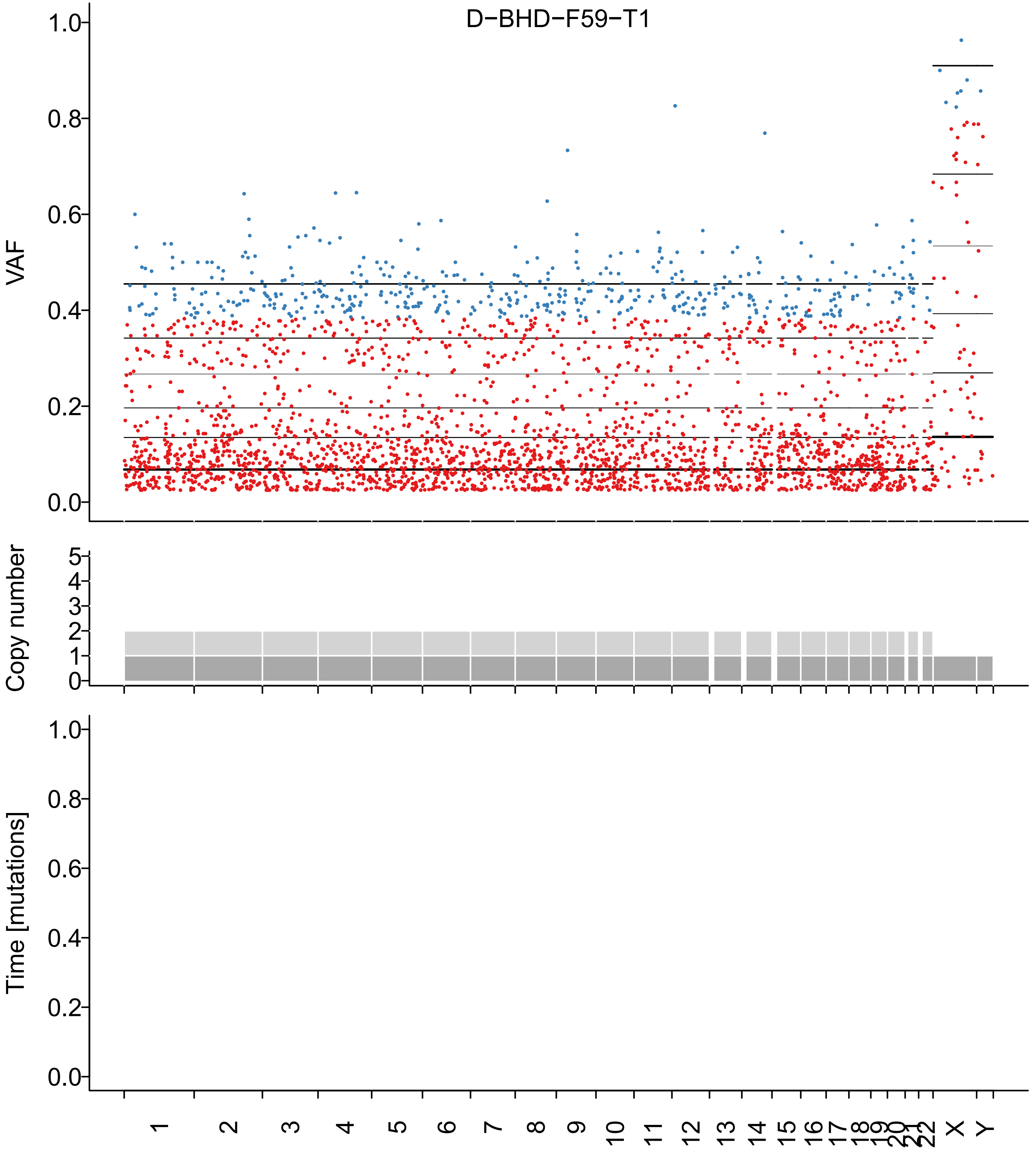

D−BHD−F59−T1
VAF
1.0
0.8
0.6
0.4
0.2
0.0
Copy number
5
4
3
2
1
0
Time [mutations]
1.0
0.8
0.6
0.4
0.2
0.0
1
2
3
4
5
6
7
8
9
10
11
12
13
14
15
16
17
18
19
20
21
22
X
Y

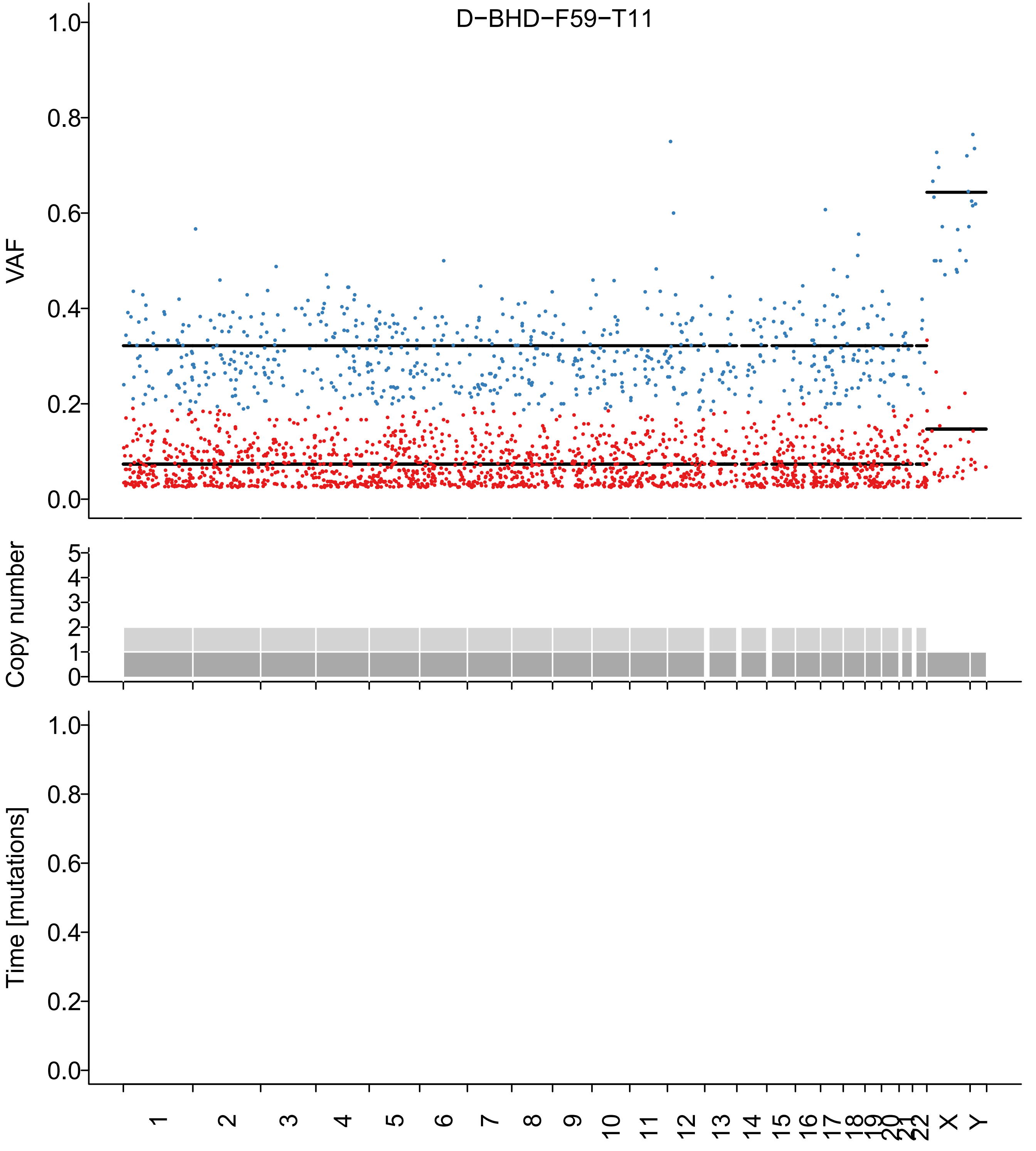

D−BHD−F59−T11
VAF
1.0
0.8
0.6
0.4
0.2
0.0
Copy number
5
4
3
2
1
0
Time [mutations]
1.0
0.8
0.6
0.4
0.2
0.0
1
2
3
4
5
6
7
8
9
10
11
12
13
14
15
16
17
18
19
20
21
22
X
Y

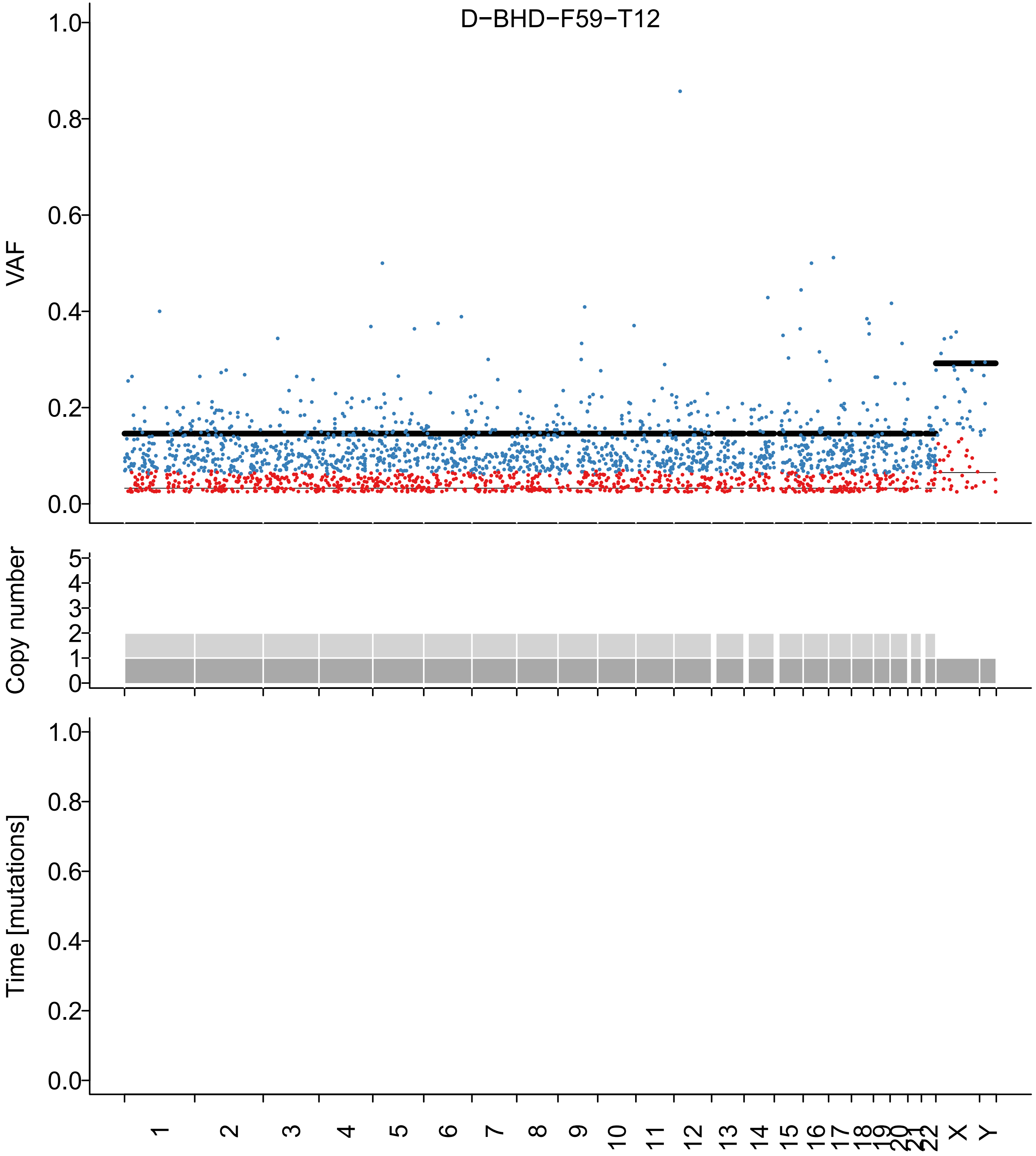

D-BHD-F59-T12
VAF
1.0
0.8
0.6
0.4
0.2
0.0
Copy number
5
4
3
2
1
0
Time [mutations]
1.0
0.8
0.6
0.4
0.2
0.0
1 2 3 4 5 6 7 8 9 10 11 12 13 14 15 16 17 18 19 20 21 22 X Y

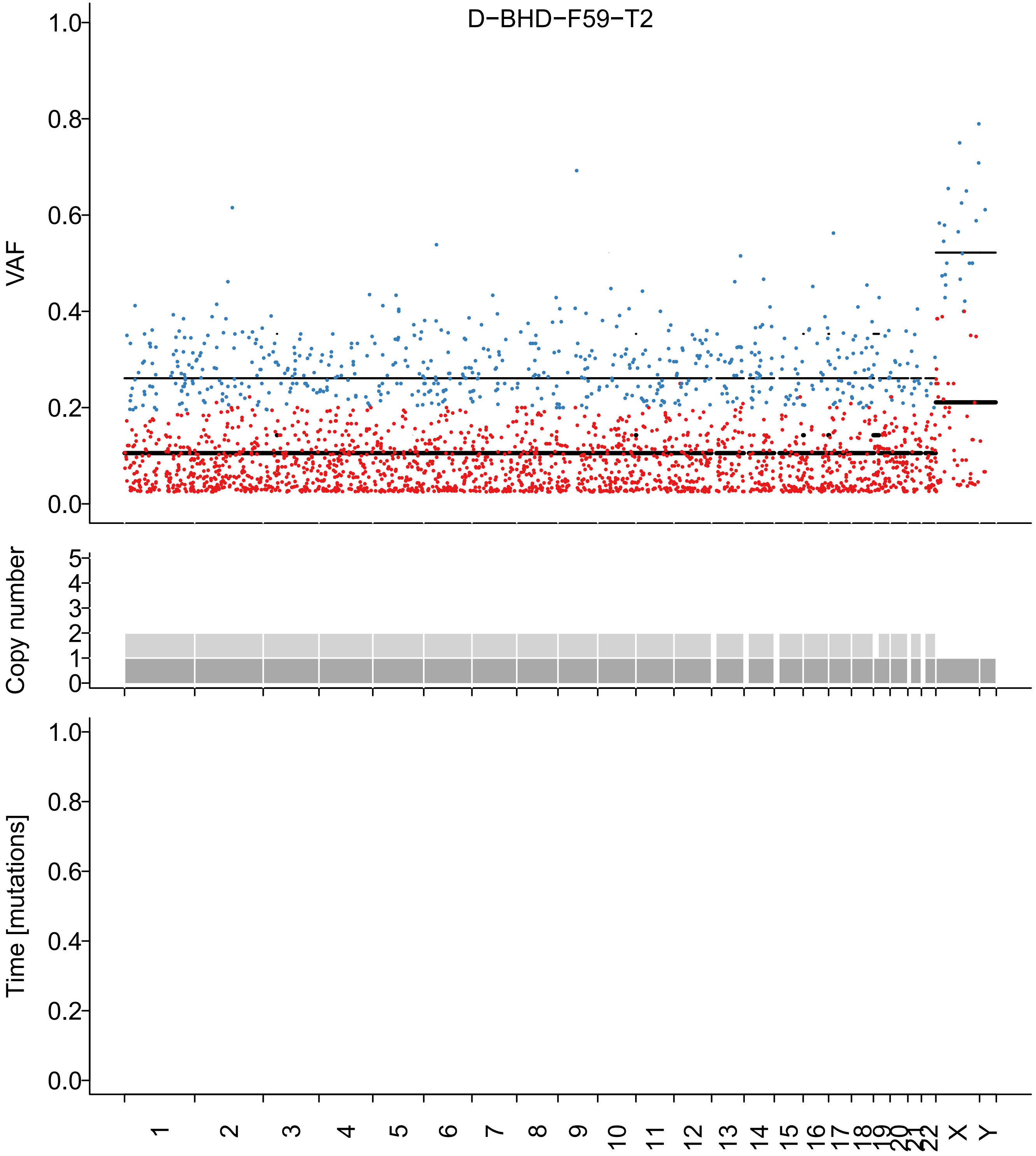
D−BHD−F59−T2
VAF
1.0
0.8
0.6
0.4
0.2
0.0
Copy number
5
4
3
2
1
0
Time [mutations]
1.0
0.8
0.6
0.4
0.2
0.0
1
2
3
4
5
6
7
8
9
10
11
12
13
14
15
16
17
18
19
20
21
22
X
Y

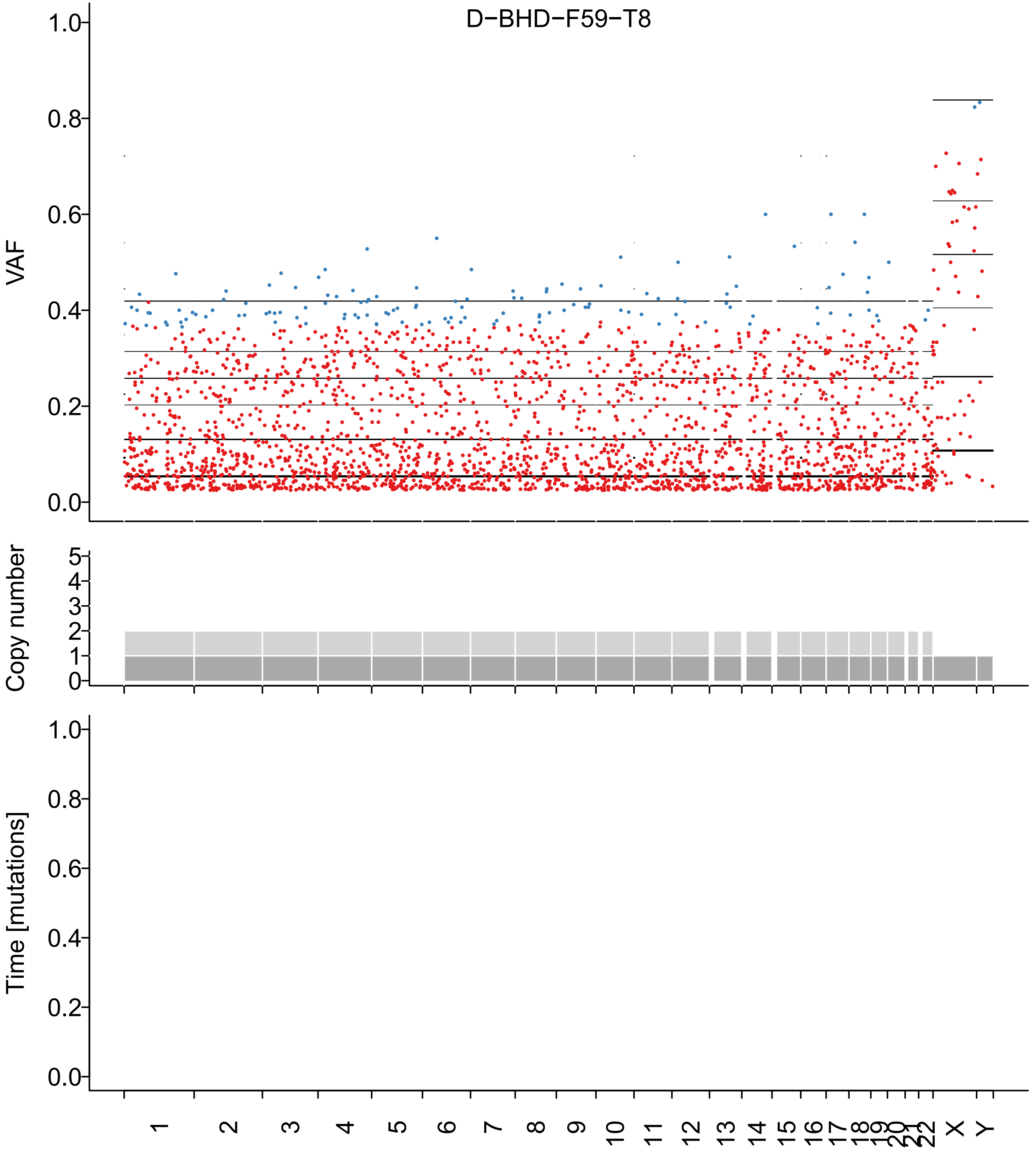

D−BHD−F59−T8
VAF
1.0
0.8
0.6
0.4
0.2
0.0
Copy number
5
4
3
2
1
0
Time [mutations]
1.0
0.8
0.6
0.4
0.2
0.0
1
2
3
4
5
6
7
8
9
10
11
12
13
14
15
16
17
18
19
20
21
22
X
Y

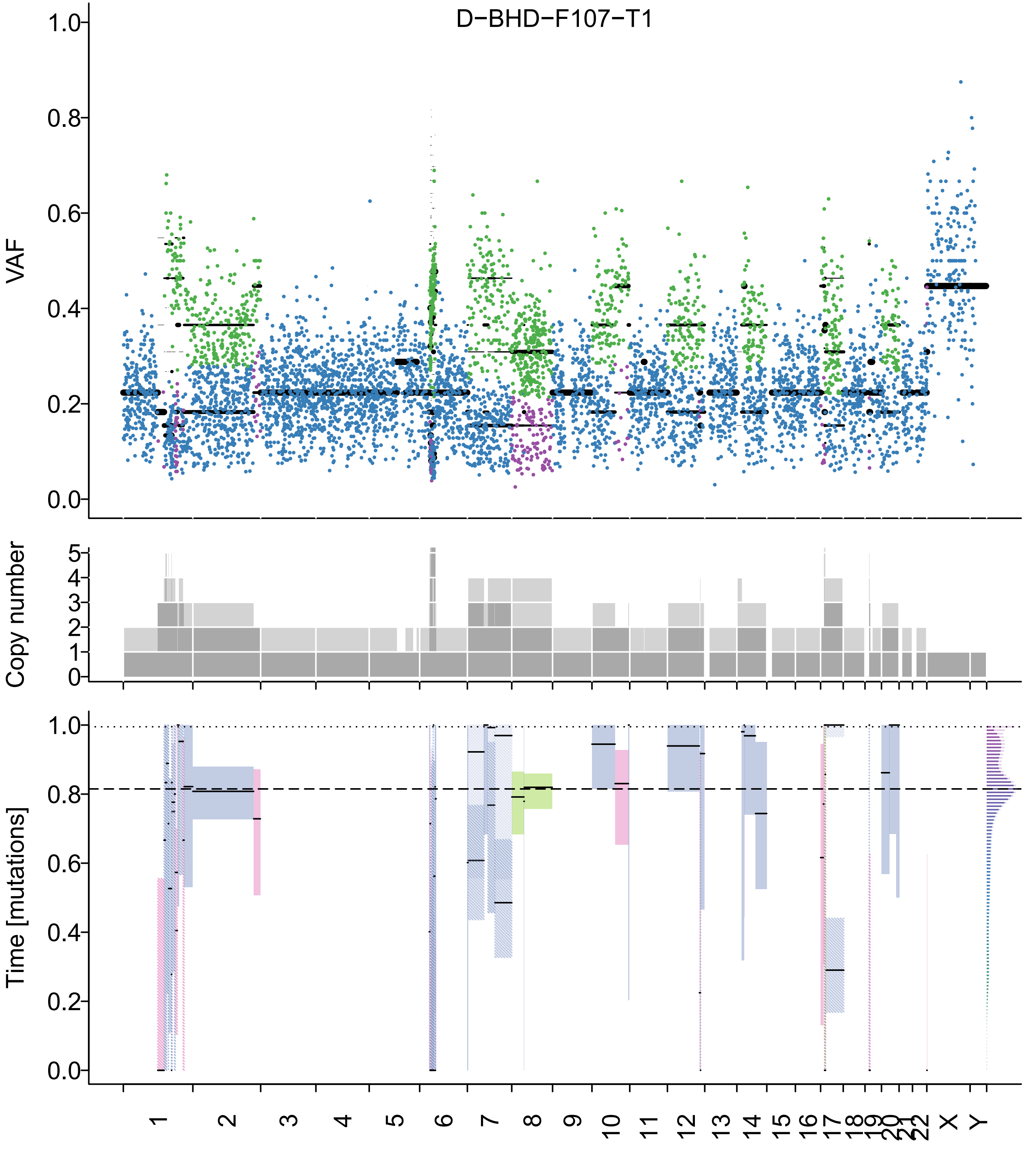
D−BHD−F107−T1
VAF
Copy number
Time [mutations]
1 2 3 4 5 6 7 8 9 10 11 12 13 14 15 16 17 18 19 20 21 22 X Y

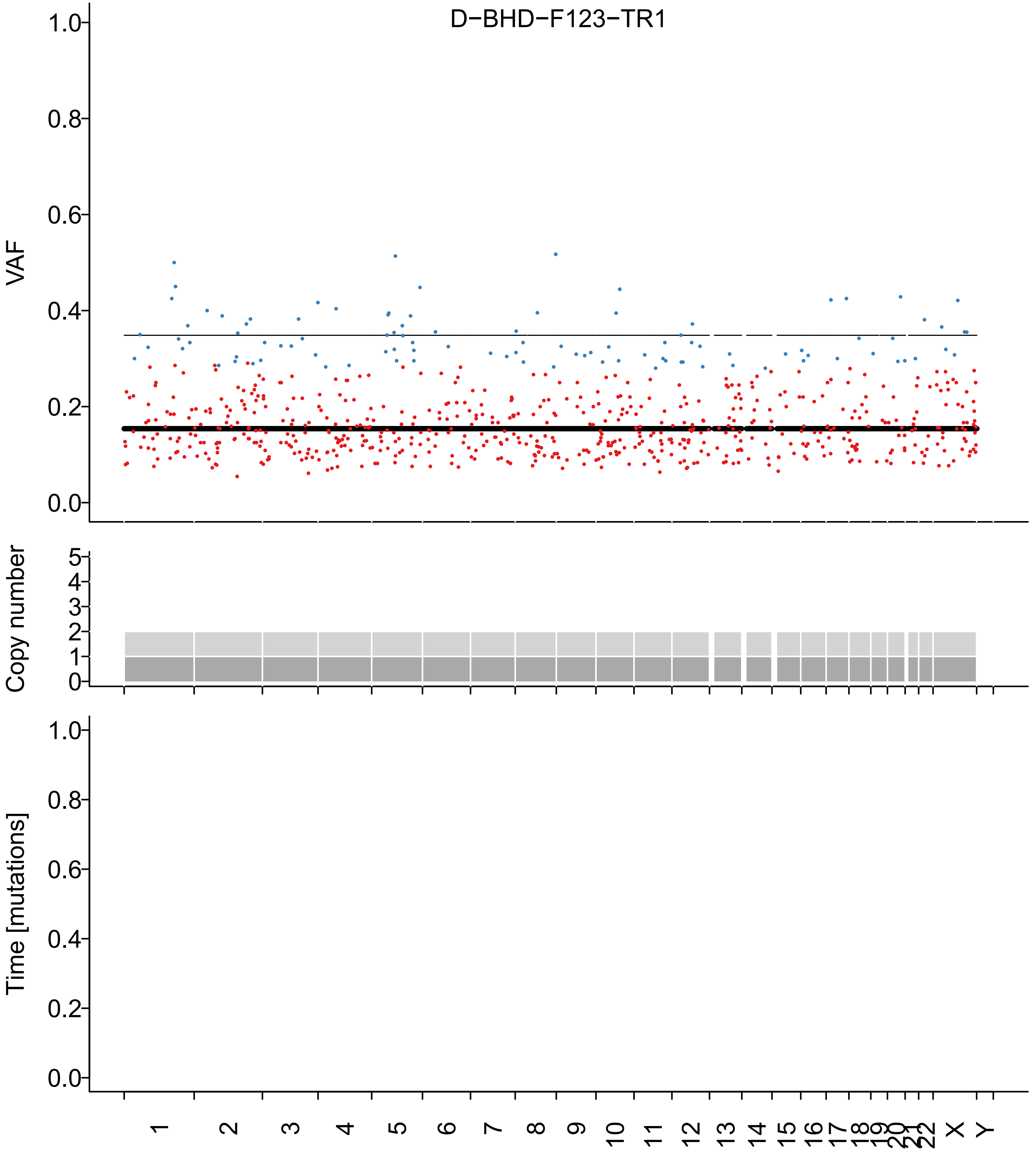
D−BHD−F123−TR1
VAF
Copy number
Time [mutations]
1
2
3
4
5
6
7
8
9
10
11
12
13
14
15
16
17
18
19
20
21
22
X
Y

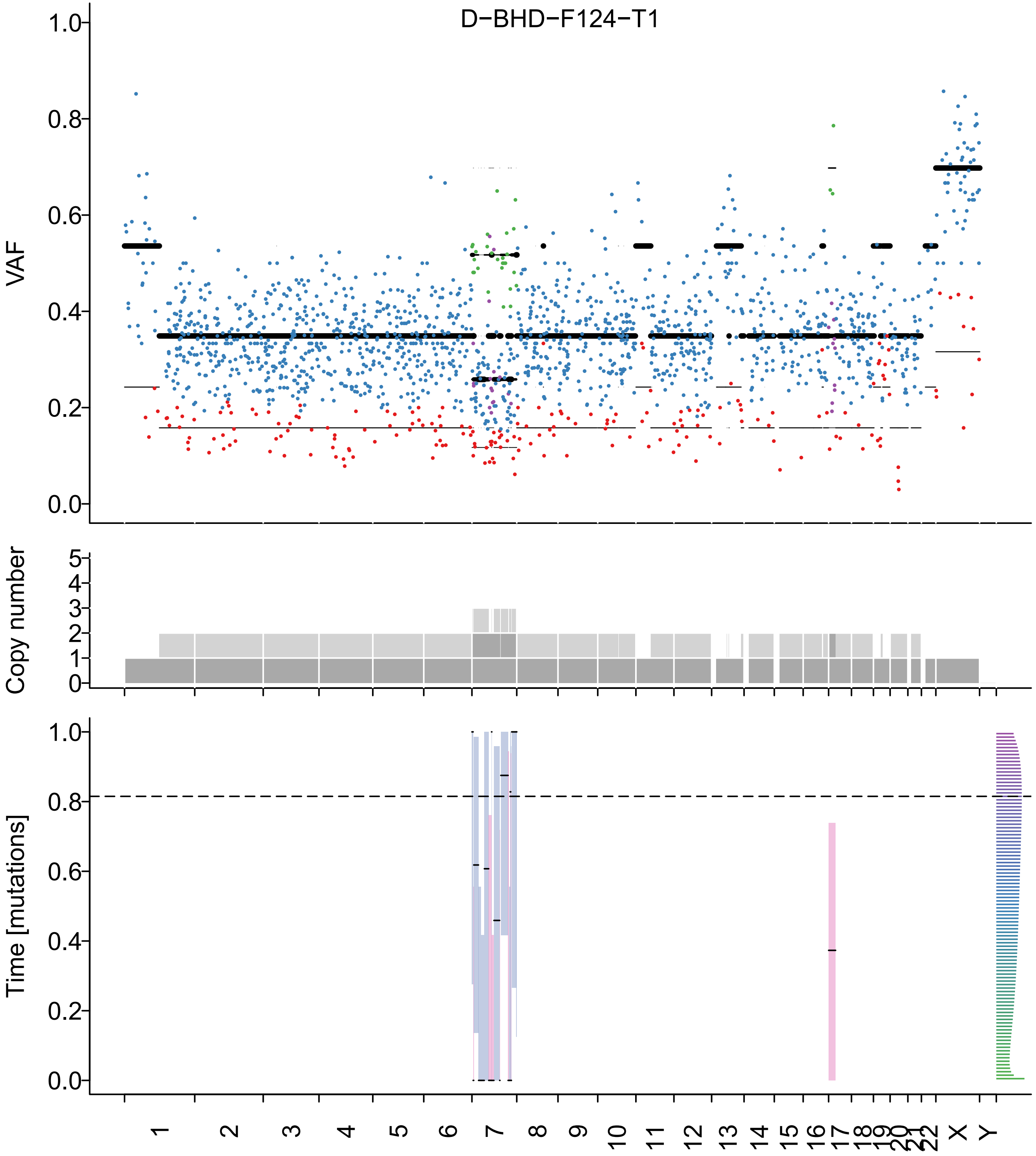
D−BHD−F124−T1
VAF
1.0
0.8
0.6
0.4
0.2
0.0
Copy number
5
4
3
2
1
0
Time [mutations]
1.0
0.8
0.6
0.4
0.2
0.0
1 2 3 4 5 6 7 8 9 10 11 12 13 14 15 16 17 18 19 20 21 22 X Y

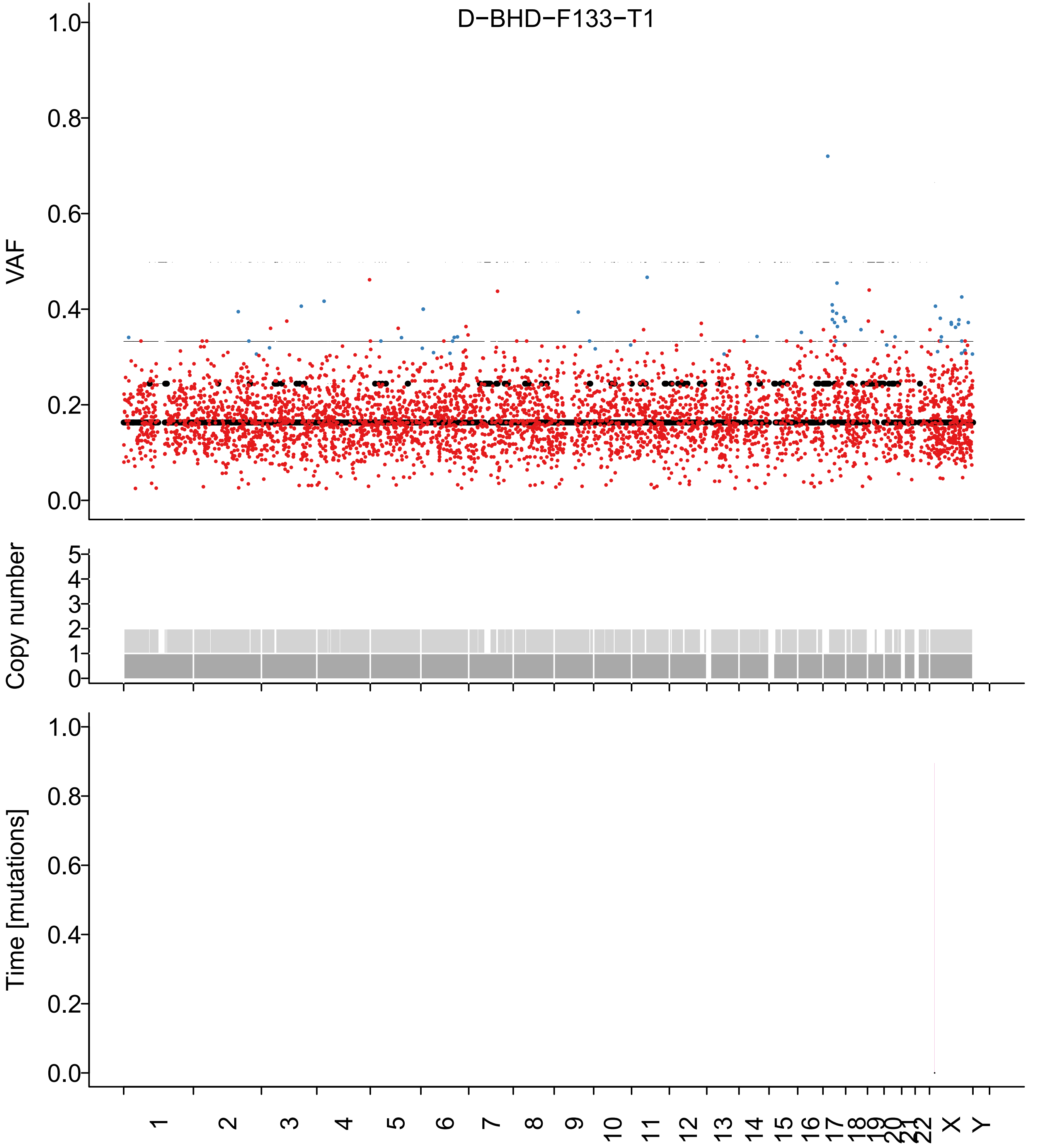
D−BHD−F133−T1
VAF
1.0
0.8
0.6
0.4
0.2
0.0
Copy number
5
4
3
2
1
0
Time [mutations]
1.0
0.8
0.6
0.4
0.2
0.0
1
2
3
4
5
6
7
8
9
10
11
12
13
14
15
16
17
18
19
20
21
22
X
Y

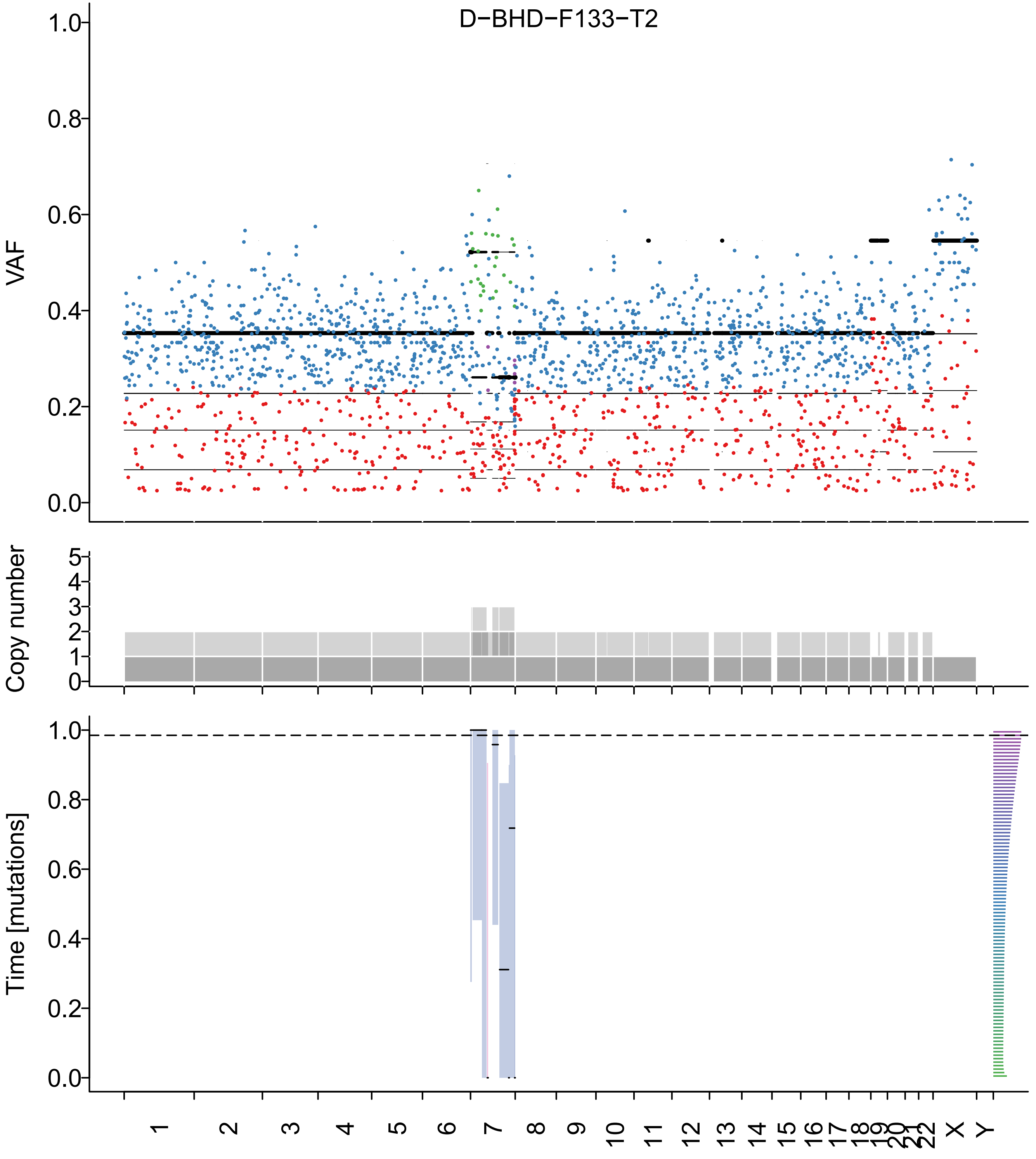

D−BHD−F133−T2
VAF
1.0
0.8
0.6
0.4
0.2
0.0
Copy number
5
4
3
2
1
0
Time [mutations]
1.0
0.8
0.6
0.4
0.2
0.0
1
2
3
4
5
6
7
8
9
10
11
12
13
14
15
16
17
18
19
20
21
22
X
Y

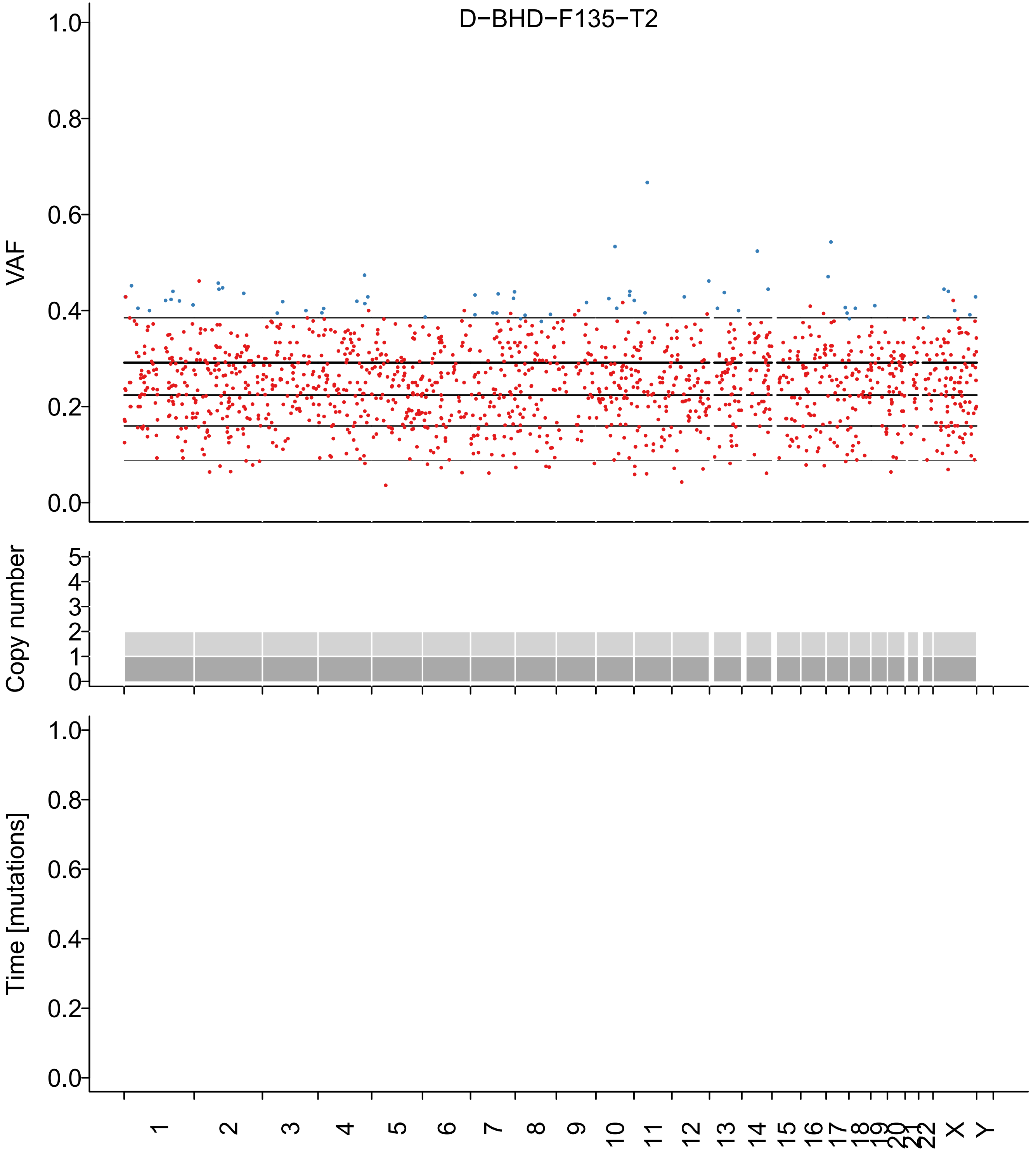
D−BHD−F135−T2
VAF
1.0
0.8
0.6
0.4
0.2
0.0
Copy number
5
4
3
2
1
0
Time [mutations]
1.0
0.8
0.6
0.4
0.2
0.0
1
2
3
4
5
6
7
8
9
10
11
12
13
14
15
16
17
18
19
20
21
22
X
Y

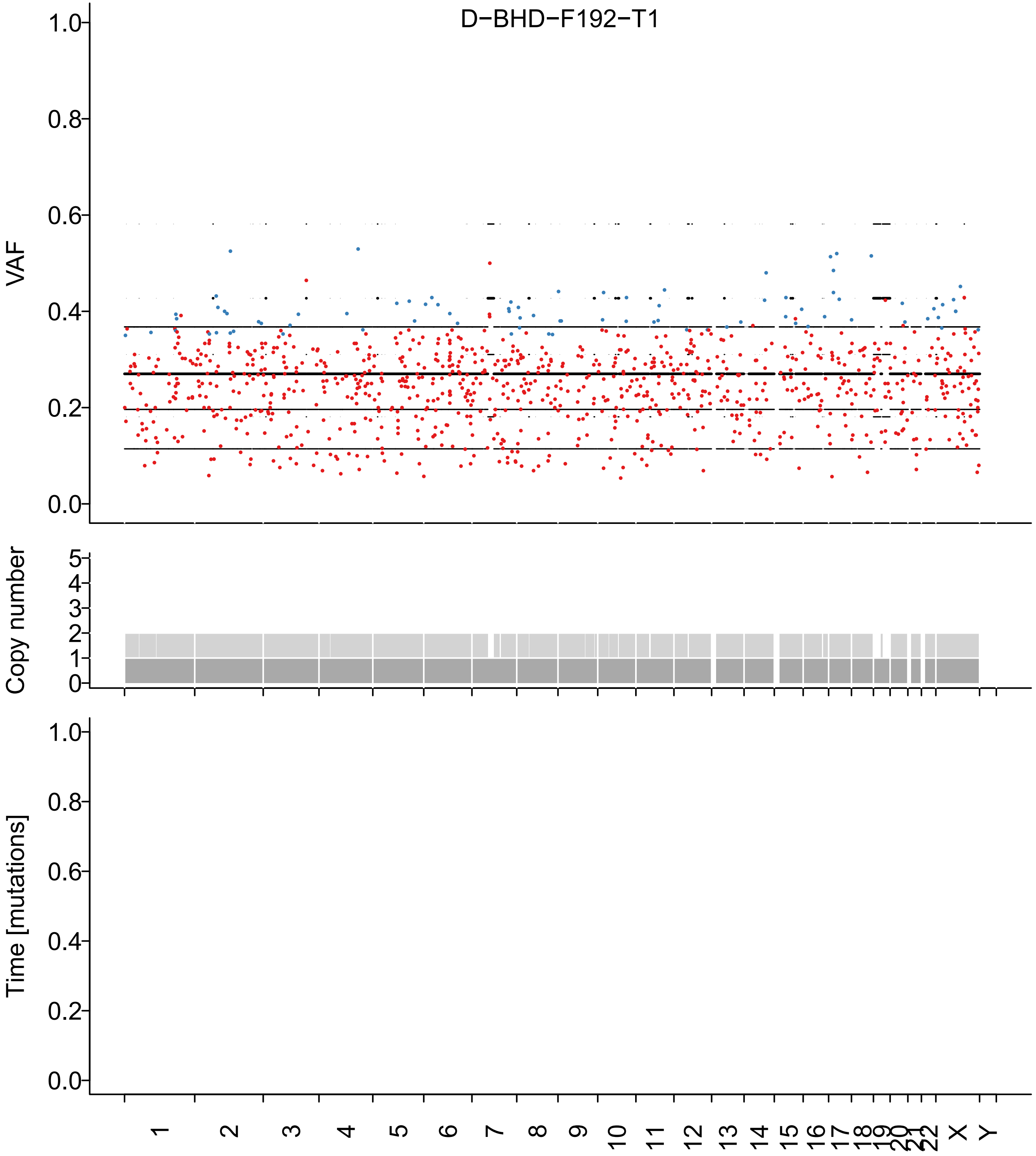

D−BHD−F192−T1
VAF
1.0
0.8
0.6
0.4
0.2
0.0
Copy number
5
4
3
2
1
0
Time [mutations]
1.0
0.8
0.6
0.4
0.2
0.0
1
2
3
4
5
6
7
8
9
10
11
12
13
14
15
16
17
18
19
20
21
22
X
Y
