# Supplementary material for: Comparative analyses define differences between BHD-associated renal tumour and sporadic chromophobe renal cell carcinoma
Source: eBioMedicine. 2023 May 12;92:104596. doi: 10.1016/j.ebiom.2023.104596 (PMC10200853; doi:10.1016/j.ebiom.2023.104596)
Supplement: Caption for supplementary material [file mmc14.docx]

**Supplementary Figure Legends**

**Supplementary Figure 1. Sporadic tumors formed two distinct subclusters characterized by *L1CAM* and *FOXI1* expressions. Rerated to Figure 2.**

(a) Violin plots show *L1CAM* and *FOXI1* expressions in each cell cluster of BHD-associated ChRCC and BHD-associated HOCT depicted in Figure 2c.

(b) Line plot shows the expression levels of *FOXI1* and *L1CAM* in sixteen BHD-associated renal tumors, twenty-one sporadic ChRCCs and seven sporadic oncocytomas (upper panel). Line plot shows the expression levels of *FOXI1* and *L1CAM* in forty-nine sporadic ChRCCs in TCGA cohort (KICH) (lower panel).

**Supplementary Figure 2. Correlation between age and number of mutations in tumor samples and structural variants in BHD-associated renal tumor. Related to Figure 3.**

(a) Scatter plots show correlation between age and number of mutations in tumor samples. For BHD-associated renal tumor (ChRCC and HOCT), the correlation was weakly positive (R=0.39 for Pearson's correlation coefficient) but not statistically significant (p=0.17). For KICH, the correlation was weakly positive (R=0.35) and statistically significant (p=0.018) (left panel). Box plot shows the age distribution of BHD patients and patients in KICH cohort. There is no difference in age between these two groups. Pearson's correlation coefficient (right panel).

(b) Circus plots show structure variations (SV) in BHD-associated renal tumors.

(c) Heatmap shows copy number alterations (CNA) in BHD-associated renal tumors.

**Supplementary Figure 3. BHD-associated renal tumor demonstrates unique mutational signatures.**

Bar graphs and box plots show mutational signatures of single base substitutions (SBS), doublet base substitutions (DBS) and insertion and deletions (ID)of sixteen BHD-associated renal tumors and forty-nine sporadic ChRCCs in TCGA cohort (KICH). Welch's two sample t-test was used.

**Supplementary Figure 4. Mitochondrial DNA copy number in each histological type of BHD-associated renal tumor. Related to Figure 4.**

Box plots show mitochondrial DNA copy number in each histological type of BHD-associated renal tumor and their adjacent normal kidneys (Paired normal). BHD-Unclassified: BHD-associated unclassified renal cell carcinoma.

**Supplementary Figure 5. MutationTimeR results of all of the BHD-associated renal tumors.**

The plots shown are the outputs of MutationTimeR of all of the BHD-associated renal tumors. The top plots show the observed and expected variant allele frequencies of point mutations. Colors indicate the timing category: Blue = clonal [other], purple = clonal [late], green = clonal [early], red = subclonal. The middle plots show the copy number as stacked barplots. Subclonal CN is indicated by fractional bars. Dark grey is major, light grey minor allele. The bottom plots show the estimated mutation time of primary and secondary gains (shaded). Boxes denote 95% CIs. The histogram at the right shows the distribution of timing events. Blue = mono-allelic gains (N:1), pink = CN-LOH/gain+loss (N:0) and green = bi-allelic gains (N:2). Among these results, F42-T1 and F124-T1 are shown in Figure 5a as samples in which the timings of *FLCN* second hit were detected.

**Supplementary Figure 6. Correlation of *L1CAM* or *FOXI1* expression with Notch signaling-mediated network expression.**

Scatter plots show the correlations of *L1CAM* or *FOXI1* expressions and Notch signaling-mediated network expression. Pearson's correlation coefficient and p-value are shown.
